# A KMT2A-AFF1 gene regulatory network highlights the role of core transcription factors and reveals the regulatory logic of key downstream target genes

**DOI:** 10.1101/gr.268490.120

**Published:** 2021-07

**Authors:** Joe R. Harman, Ross Thorne, Max Jamilly, Marta Tapia, Nicholas T. Crump, Siobhan Rice, Ryan Beveridge, Edward Morrissey, Marella F.T.R. de Bruijn, Irene Roberts, Anindita Roy, Tudor A. Fulga, Thomas A. Milne

**Affiliations:** 1 MRC Molecular Haematology Unit, MRC Weatherall Institute of Molecular Medicine, Radcliffe Department of Medicine, University of Oxford, Oxford, OX3 9DS, United Kingdom;; 2 MRC Weatherall Institute of Molecular Medicine, Radcliffe Department of Medicine, University of Oxford, Oxford, OX3 9DS, United Kingdom;; 3 MRC Molecular Haematology Unit, MRC Weatherall Institute of Molecular Medicine, Department of Paediatrics, University of Oxford, Oxford, OX3 9DS, United Kingdom;; 4 Virus Screening Facility, MRC Weatherall Institute of Molecular Medicine, John Radcliffe Hospital, University of Oxford, Oxford, OX3 9DS, United Kingdom;; 5 Center for Computational Biology, Weatherall Institute of Molecular Medicine, University of Oxford, John Radcliffe Hospital, Oxford OX3 9DS, United Kingdom;; 6 NIHR Oxford Biomedical Research Centre Haematology Theme, University of Oxford, Oxford, OX3 9DS, United Kingdom

## Abstract

Regulatory interactions mediated by transcription factors (TFs) make up complex networks that control cellular behavior. Fully understanding these gene regulatory networks (GRNs) offers greater insight into the consequences of disease-causing perturbations than can be achieved by studying single TF binding events in isolation. Chromosomal translocations of the *lysine methyltransferase 2A* (*KMT2A*) gene produce KMT2A fusion proteins such as KMT2A-AFF1 (previously MLL-AF4), causing poor prognosis acute lymphoblastic leukemias (ALLs) that sometimes relapse as acute myeloid leukemias (AMLs). KMT2A-AFF1 drives leukemogenesis through direct binding and inducing the aberrant overexpression of key genes, such as the anti-apoptotic factor *BCL2* and the proto-oncogene *MYC*. However, studying direct binding alone does not incorporate possible network-generated regulatory outputs, including the indirect induction of gene repression. To better understand the KMT2A-AFF1-driven regulatory landscape, we integrated ChIP-seq, patient RNA-seq, and CRISPR essentiality screens to generate a model GRN. This GRN identified several key transcription factors such as RUNX1 that regulate target genes downstream of KMT2A-AFF1 using feed-forward loop (FFL) and cascade motifs. A core set of nodes are present in both ALL and AML, and CRISPR screening revealed several factors that help mediate response to the drug venetoclax. Using our GRN, we then identified a KMT2A-AFF1:RUNX1 cascade that represses *CASP9*, as well as KMT2A-AFF1-driven FFLs that regulate *BCL2* and *MYC* through combinatorial TF activity. This illustrates how our GRN can be used to better connect KMT2A-AFF1 behavior to downstream pathways that contribute to leukemogenesis, and potentially predict shifts in gene expression that mediate drug response.

The regulated transcription of genes in eukaryotes is a core aspect of cell behavior. Although some individual genes are critically required for normal tissue development, these processes, and homeostasis, are driven by the coordinated regulation of entire sets of genes (Reik 2007; Baxter et al. 2014). Disruption of these genes can lead to abnormal development and human diseases such as leukemia (Thoms et al. 2019). Gene regulatory patterns are controlled by transcription factors (TFs), that often function together in complex cooperative patterns that are hard to functionally dissect (Bhattacharjee et al. 2013; Reiter et al. 2017). To understand normal and aberrant cell behavior, better models of this combinatorial code are needed.

To understand this combinatorial code, researchers have constructed gene regulatory networks (GRNs) modeling developmental systems, such as hematopoietic specification (Goode et al. 2016), T-lymphocyte specification (Georgescu et al. 2008), neural crest development (Williams et al. 2019), and cancers, including lymphoma (de Matos Simoes et al. 2013) and leukemia (Assi et al. 2019). A challenging step is to use a GRN to predict key regulatory interactions, potentially by breaking it down into simple three-node motifs. Motifs include the feed-forward loop (FFL), which is enriched in biological GRNs (Milo et al. 2002; Mangan and Alon 2003), and the TF cascade (or regulator chain), in which intermediate TFs bridge to indirect targets (Lee et al. 2002; Rosenfeld and Alon 2003). Although motifs are compelling, these patterns do not always elucidate biological function (Ingram et al. 2006) and are difficult to use to predict disease prognosis or progression, for instance. For these reasons, GRNs are better considered as a collection of predictive pathways and potential cooperative interactions that need to be experimentally validated.

Leukemias driven by rearrangements of the *lysine methyltransferase 2A* (*KMT2A*, previously known as *MLL*) gene do not respond well to treatment and have a very poor prognosis (Krivtsov and Armstrong 2007; Milne 2017). *KMT2A* rearrangements (*KMT2A*r) are chromosome translocations that fuse *KMT2A* in frame to one of a wide number of partner genes, creating novel fusion proteins (KMT2A-FPs), the most common of which is KMT2A-AFF1 (formerly MLL-AF4 or MLL-MLLT2) (Meyer et al. 2013). *KMT2A*r leukemias have few cooperating mutations (Bardini et al. 2010, 2011; The Cancer Genome Atlas Research Network 2013; Andersson et al. 2015), thus the KMT2A-FP is able to drive leukemogenesis alone through aberrant gene expression profiles. KMT2A-AFF1 is most commonly associated with acute lymphoblastic leukemia (ALL) (Meyer et al. 2013) but can display a mixed phenotype with features of both acute myeloid leukemia (AML) and ALL (Alexander et al. 2018). A subset of ALLs, such as KMT2A-AFF1, can also relapse after treatment to become an AML derived from the original leukemic clone (Dorantes-Acosta and Pelayo 2012; Gardner et al. 2016; Pillai et al. 2019), which is indicative of a core KMT2A-AFF1 GRN that can drive both ALL and AML.

KMT2A-FPs promote transcription by recruiting a large transcription elongation complex to target genes (Ballabio and Milne 2012; Slany 2020; Takahashi and Yokoyama 2020). Multiple directly bound KMT2A-AFF1 targets are crucial for driving leukemogenesis, including *BCL2* and TFs such as *MYC* and *RUNX1* (Guenther et al. 2008; Wilkinson et al. 2013; Benito et al. 2015). Because of this, much emphasis has been placed on therapeutically targeting individual gene products, but monotherapies are often susceptible to relapse and resistance, even when preclinical models show initial sensitivity to targeted therapies such as the BCL2 protein inhibitor venetoclax (Niu et al. 2014; Benito et al. 2015; Khaw et al. 2016). Understanding the regulation of genes such as *MYC* and *BCL2*, and more broadly growth and apoptosis pathways, is key to understanding leukemic behavior and may also inform the ways in which leukemias acquire resistance.

Because *KMT2A*r leukemias have very few cooperating mutations (Bardini et al. 2010, 2011; Andersson et al. 2015), they represent an ideal system for understanding how a single perturbation can drive a leukemic GRN. This study aims to integrate RNA-seq and ChIP-seq data to create a GRN that includes both directly bound targets of KMT2A-AFF1 as well as the broader network of indirect targets (Fig. 1A). Using this model to construct testable hypotheses, we further aim to elucidate key FFL and cascade network motifs that drive leukemogenesis.

**Figure 1.**
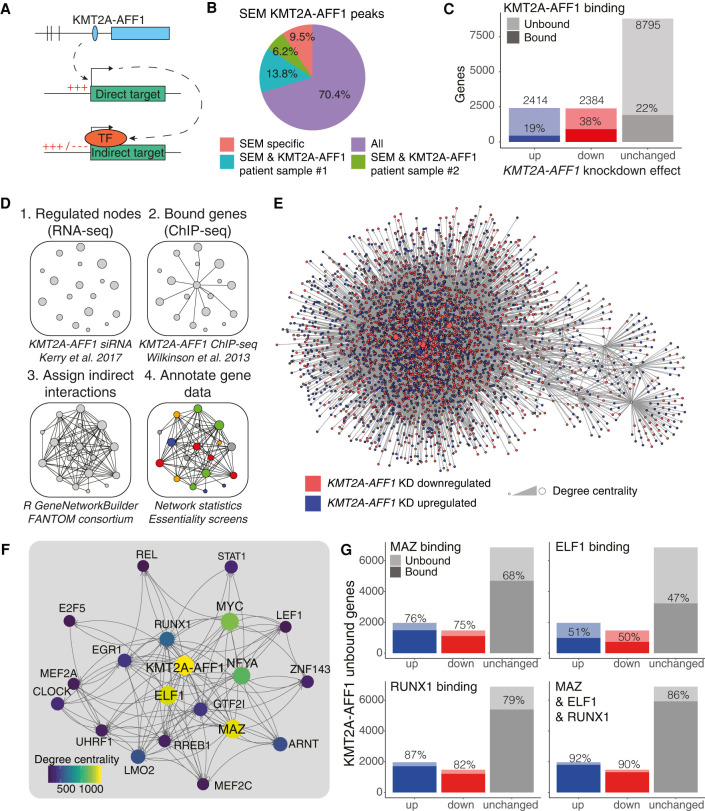
Developing a GRN model to assess regulatory impact of KMT2A-AFF1. (*A*) Schematic illustrating the concept of KMT2A-AFF1-targeted TFs, leading to indirect downstream regulation. (*B*) Proportion of KMT2A-AFF1-bound genes in SEM cells (nearest annotated promoter in ChIP-seq) that overlap with KMT2A-AFF1 targets from patient samples. (*C*) DEGs from nascent RNA-seq after 96 h *KMT2A-AFF1* KD. DEGs are defined as FDR < 0.05 (*n* = 3). Shaded area represents KMT2A-AFF1-bound genes. (*D*) GRN creation workflow using nascent RNA-seq and ChIP-seq data (Methods). (*E*) Visualization of the whole network. Node color represents down-regulation (red) and up-regulation (blue) upon *KMT2A-AFF1* KD. (*F*) The top 20 genes of the KMT2A-AFF1 GRN by degree centrality. Lines indicate predicted interaction from protein to gene locus, with arrowheads pointing downstream. (*G*) *KMT2A-AFF1* KD DEGs that are unbound by KMT2A-AFF1, as highlighted in *C*. Shaded areas represent MAZ, ELF1, or RUNX1-bound genes.

## Results

### The KMT2A-AFF1 fusion protein controls a wider gene network via key TFs

We previously characterized the genome-wide binding of KMT2A-AFF1 (Kerry et al. 2017) in SEM cells, a patient-derived B-ALL KMT2A-AFF1 cell line (Greil et al. 1994). We annotated KMT2A-AFF1 ChIP-seq peaks with the nearest gene promoter (Wilkinson et al. 2013), which we refer to as KMT2A-AFF1-bound genes. As immortalized cell lines undergo transcriptional adaptations (Lopes-Ramos et al. 2017), we validated our KMT2A-AFF1-bound genes with ChIP-seq in two different KMT2A-AFF1 ALL patient samples. The majority of peaks are common (70%) (Fig. 1B), and the level of KMT2A-AFF1 bound at promoters is well correlated between samples (Supplemental Fig. S1A). Some of the differences in bound genes between the data sets are likely a result of variable peak calling, because there is observable KMT2A-N signal in all samples at “uniquely bound” genes (Supplemental Fig. S1B,C). Peak calling issues can be caused by a sonication bias toward regions of open chromatin (Landt et al. 2012), creating “peaks” in the input tracks, but our SEM data showed minimal such bias (Supplemental Fig. S1D). Taken together, this analysis suggests that SEM cells are a reasonable model of KMT2A-AFF1 binding in patients.

Considering the transcription activating role of the KMT2A-AFF1 complex (Mueller et al. 2007; Lin et al. 2010; Yokoyama et al. 2010; Biswas et al. 2011; Ballabio and Milne 2012; Okuda et al. 2015; Kerry et al. 2017), we expected that following *KMT2A-AFF1* siRNA knockdown (KD) (Kerry et al. 2017), differentially expressed genes (DEGs) would be biased toward down-regulation. Instead, up- and down-regulated genes showed no bias in either direction (Fig. 1C). KMT2A-AFF1 is considered to be the main driver of leukemogenesis, therefore we expected the majority of DEGs would be directly bound by KMT2A-AFF1. Instead, only 19% of up-regulated and 38% of down-regulated genes are bound (Fig. 1C). We hypothesized that the majority of these unbound DEGs may be regulated by intermediate TFs whose expression is regulated by KMT2A-AFF1 (Fig. 1A).

To explore this possibility, we constructed a KMT2A-AFF1 GRN (Methods) by integrating *KMT2A-AFF1* KD DEGs, SEM KMT2A-AFF1 ChIP-seq, and published TF interaction data (Fig. 1D,E; Supplemental Data S1; The FANTOM Consortium and the Riken Omics Science Center 2009). The top nodes of this GRN, when ranked by degree centrality (number of connections to and from a node) include TFs such as *ELF1*, *MAZ*, and *RUNX1* (Fig. 1F). We have previously shown a role for ELF1 and RUNX1 in KMT2A-AFF1 leukemias (Wilkinson et al. 2013; Godfrey et al. 2019). ChIP-seq data for ELF1, RUNX1, and MAZ revealed binding of these TFs at the majority of non-KMT2A-AFF1-bound DEGs (Fig. 1G). Combining these binding patterns has the capacity to account for >90% of *KMT2A-AFF1* KD DEGs (Fig. 1G). These data suggest that a complex interplay of multiple TFs may determine the expression profile of most GRN genes, providing potential mechanisms by which KMT2A-AFF1 could regulate indirect targets.

To test the robustness of the core nodes of the SEM GRN, we created a GRN model using a different KMT2A-AFF1 ALL cell line, RS4;11, using the same methodology (Fig. 1D). The *KMT2A-AFF1* siRNA for RS4;11 cells was considerably less efficient than the SEM siRNA (Supplemental Fig. S2A), as has been previously observed (Thomas et al. 2005; Geng et al. 2012), but many of the most central nodes of the SEM KMT2A-AFF1 network, including *ELF1*, *MYC*, and *RUNX1* are present in both GRNs (Supplemental Fig. S2B–D). We additionally compared the SEM GRN with networks centered around KMT2A-AFF1 complex associated factors, DOT1 like histone lysine methyltransferase (DOT1L) (Lacoste et al. 2002; Milne et al. 2005; Okada et al. 2005; Mueller et al. 2007; Krivtsov et al. 2008; Bernt et al. 2011; Biswas et al. 2011; Lin et al. 2016; Slany 2020) and BRD4 (Supplemental Fig. S2E; Dawson et al. 2011; Zuber et al. 2011). These networks show considerable overlap with the SEM KMT2A-AFF1 GRN, albeit not fully with the central GRN TFs (Supplemental Fig. S2F–H).

### ALL and AML patient subnetworks highlight a set of core TFs

**Figure 2.**
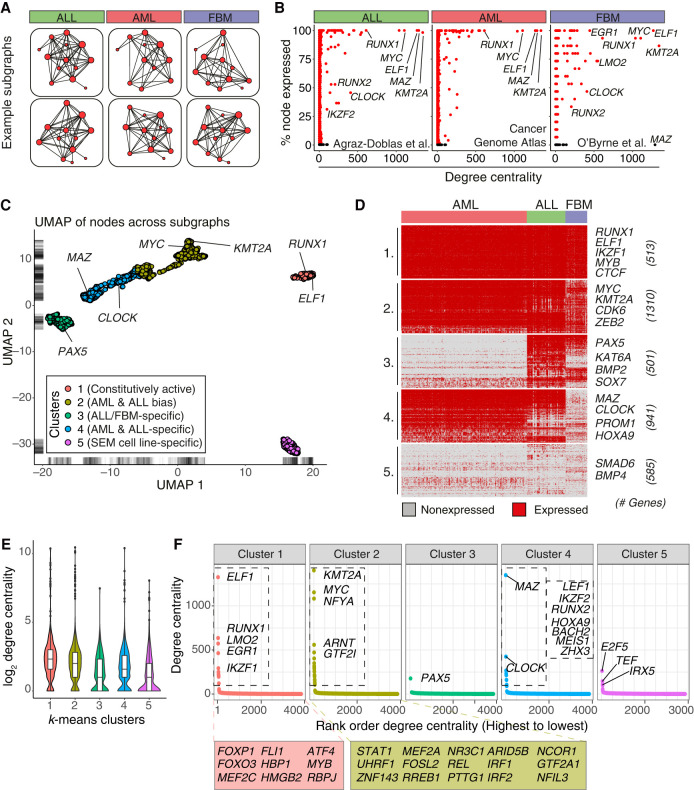
Patient subnetwork analysis highlights core KMT2A-FP GRN circuitry. (*A*) Subnetworks reflecting expressed genes in patient samples. (*B*) Percentage of samples in each RNA-seq data set that express each gene plotted against degree centrality in the KMT2A-AFF1 GRN. (*C*) UMAP dimensionality reduction of node activity across subnetworks, colored according to *k*-means clusters. (*D*) Heatmap of node activity across subnetworks, separated into *k*-means clusters as in *C*. Rows represent genes; columns represent samples. Red and gray indicates gene expression above and below the mean, respectively. (*E*) Violin and box plots showing distribution of log_2_ degree centrality of GRN nodes, stratified by *k*-means cluster. Box plot midline indicates median, and *upper* and *lower* hinges indicate 1st and 3rd quartiles, respectively. Whiskers extend to furthest values within 1.5 times the interquartile range. (*F*) Degree centrality of nodes plotted against rank order of centrality. Annotated nodes in Clusters 1, 2, and 4 represent the core TFs of the GRN model.

To test the applicability of our model to leukemia in patients, we came up with a strategy to integrate patient data into our GRN (Fig. 2A) to answer three questions. First, how much of the GRN is conserved in *KMT2A*r ALL patients? Second, is there a core program conserved across acute leukemias, including AML? Finally, how much of the core GRN represents pathways that have been co-opted from normal hematopoietic cell populations? We used RNA-seq from *KMT2A*r ALL patients (Agraz-Doblas et al. 2019), AML patients with a range of chromosomal abnormalities (The Cancer Genome Atlas Research Network 2013), and hematopoietic stem and progenitor cell (HSPC) populations and B cells from normal fetal bone marrow (FBM) (O'Byrne et al. 2019) to generate individual patient-specific subnetworks derived from our SEM KMT2A-AFF1 GRN (Supplemental Fig. S3A; Methods).

The most central nodes of the KMT2A-AFF1 GRN (degree centrality > 500, 8/3850 nodes) are constitutively active across ALL and AML patient subnetworks (Fig. 2B) and are bound by KMT2A-AFF1 in the ALL patient ChIP-seq samples, indicating that they are commonly up-regulated by KMT2A-AFF1 in leukemia (Supplemental Fig. S3B). Because the AML data set contains some non-*KMT2A*r leukemias, these factors may apply more generally to leukemia biology. Node conservation was reduced in the FBM samples compared to the leukemia data sets, implying that the circuitry of the GRN differs from normal hematopoietic cell behavior (Fig. 2B). For example, *LMO2* is expressed in only 75%, and *MAZ* is inactive in all FBM samples. This is consistent with the concept that KMT2A-AFF1 drives the GRN to increase, or sustain activation of, TF regulatory motifs. Our patient data analysis does not account for levels of expression, and cases of overexpression or subtle changes are not captured. For example, *RUNX1* is active in both leukemias and FBM, but past work has also shown that *RUNX1* expression is higher in *KMT2A-AFF1* ALLs than in *KMT2A-MLLT3* AML (Wilkinson et al. 2013). This overexpression of FBM-expressed factors implies that KMT2A-FPs also serve to co-opt normal hematopoietic circuits. These observations suggest that although the core GRN motifs represent common regulatory wiring that enables an oncogenic transcription program, levels of gene and protein expression likely also contribute to oncogenesis.

To explore the primary RNA-seq data in detail, we clustered node activity patterns into five groups (Fig. 2C,D; Supplemental Data S2). Clusters 1 and 2 are active across all data sets and includes *RUNX1*, *ELF1, KMT2A*, and *MYC*; Cluster 1 is enriched for hematopoietic differentiation processes (Supplemental Fig. S3C). In the case of *KMT2A*r leukemias, *KMT2A* will also represent KMT2A-FP activity. Cluster 3 contains ALL- and FBM-biased genes, including *SOX7* and *KAT6A* (ALL specific), and *H2AC12* (previously known as *HIST1H2AH*) and *KAT7* (ALL and FBM specific). Cluster 4 is specific to AML and ALL data sets with low activity across all FBM cell types (Supplemental Fig. S3D) and includes *MAZ* and *PROM1*. Cluster 5 is inactive in all of the data sets, and pathway enrichment implies this represents an immortalized cell program (e.g., cell junction organization) (Supplemental Fig. S3C).

Clusters 1, 2, and 4 are of particular interest because these represent commonality across acute leukemias and together contain the most central GRN nodes (Fig. 2E,F). As KMT2A-FP leukemias are capable of lineage switching (Dorantes-Acosta and Pelayo 2012; Gardner et al. 2016), this core circuitry could enable this switch. To reinforce this concept, we compared KMT2A-FP binding in SEM and THP-1 cells (a KMT2A-MLLT3 AML cell line), and found similar binding profiles (Supplemental Fig. S4A,B). Many of the core GRN nodes are bound by KMT2A-FPs in both SEM and THP-1 cells (Supplemental Fig. S4C), which suggests these central TFs represent core KMT2A-FP behavior. To further this analysis, we focused on RUNX1, a core GRN factor expressed in both AML and ALL samples (Fig. 2D, Cluster 1). Using RUNX1 ChIP-seq, we found similar overlaps in RUNX1-bound genes between SEM and THP-1 cells, albeit with differential enhancer usage (such as at *GFI1* and *EVI5*) (Supplemental Fig. S4D,E), and targeting of many central GRN nodes (Supplemental Fig. S4F). These analyses together describe distinct expression patterns in patients and FBM cell types, highlighting a core KMT2A-AFF1-driven network that exists across ALL and AML leukemias.

### RUNX1 is a highly central and essential node of the KMT2A-AFF1 network

To determine the functional importance of the core GRN nodes, we integrated data from published CRISPR essentiality screens. Analysis of the Project Score Cancer Dependency Map (Sanger Institute) (Behan et al. 2019) and the Avana CRISPR screen data set (DepMap, Broad Institute) (Doench et al. 2016; Meyers et al. 2017) showed that *MYC* is pan-essential, *ELF1* is essential in one cancer cell line, *RUNX1* is essential in multiple hematopoietic cancer cells in the DepMap data set, and *MAZ* and *ELF1* were not reported to be essential in any hematopoietic cancer models (Supplemental Fig. S5A,B).

**Figure 3.**
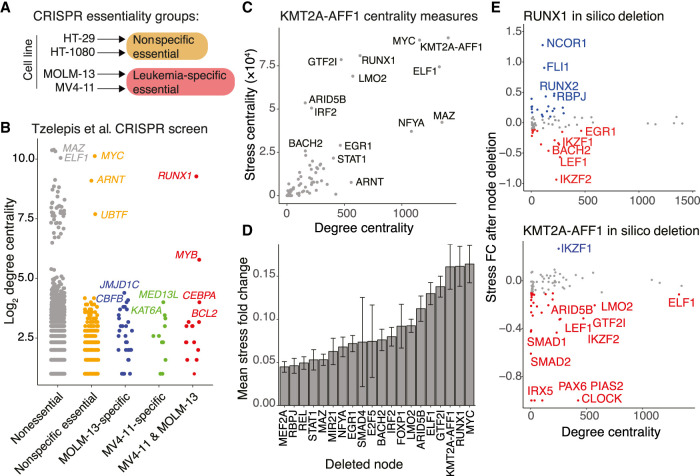
RUNX1 is a key essential node in the core KMT2A-AFF1 network. (*A*) Schematic illustrating how CRISPR screen results from Tzelepis et al. (2016) are categorized. Essential genes in HT-29 or HT-1080 were nonspecific essential. Genes not essential in HT-29 or HT-1080 but essential in MOLM-13 or MV4-11 were specific to leukemia. (*B*) Association of log_2_ degree centrality (KMT2A-AFF1 GRN) with CRISPR essentiality groups as outlined in *A*. (*C*) Degree centrality plotted against stress centrality of KMT2A-AFF1 GRN nodes. (*D*) Mean absolute stress fold change (FC) response to in silico deletion of GRN nodes and subsequent recalculation of stress centrality. Stress FC is calculated on a per gene basis in the GRN before and after in silico deletion. The top 20 nodes are shown. (*E*) Stress FC after in silico deletion of RUNX1 or KMT2A-AFF1, plotted against degree. Blue and red indicate positive (FC > 0.1 FC) and negative (FC < −0.1) stress FC, respectively.

We also analyzed a screen more targeted to leukemia, involving two *KMT2A*r cell lines, MOLM-13 (KMT2A-MLLT3 AML) and MV4-11 (KMT2A-AFF1 pediatric AML) and two non-leukemic cancer cell lines, HT-29 (colon adenocarcinoma) and HT-1080 (fibrosarcoma) (Tzelepis et al. 2016). We classified genes as nonessential, nonspecific essential (hit in HT-29 or HT-1080), and essential specifically in one or both leukemia cell lines (hit in MOLM-13 or MV4-11, but not HT-29 or HT-1080) (Fig. 3A,B). *MYC* is essential not only in leukemia cell lines, but also the non-leukemia cancer models. Conversely, *MAZ* and *ELF1* were not found to be essential in the Tzelepis screen, suggesting that their targets are not important for survival or proliferation in these models. *RUNX1* is essential for both MOLM-13 and MV4-11 cell lines, and not HT-29 or HT-1080 (Fig. 3B), in alignment with the DepMap data, in which *RUNX1* essentiality is biased toward hematopoietic cell lines (Supplemental Fig. S5B). Other key leukemia-specific hits include *MYB*, *MED13L*, *HOXA10*, and the binding partner of RUNX1, *CBFB*.

Because there is a delay between activation of a gene and protein production, network path length may influence perturbation response (Aittokallio and Schwikowski 2006). Stress centrality (number of shortest paths between any two points of a network that pass through a particular node) (Ghasemi et al. 2014) may therefore be a better indicator of essentiality. MAZ is highly connected with relatively low stress, whereas RUNX1 is among the highest stress nodes despite lower connectivity than MAZ (Fig. 3C). In silico deletion of KMT2A-AFF1, RUNX1, or MYC GRN nodes, followed by recalculation of centrality, caused considerable redistribution of stress scores across nodes, greater than in silico deletion of MAZ (Fig. 3D). This may be one reason why MAZ does not come out as a key survival gene in any of the CRISPR screens. The further implication from this analysis may be that connections are rerouted through alternative TFs in the absence of key nodes. In particular, in silico deletion of RUNX1 led to increased NCOR1 and FLI1 stress, indicating they may act as alternate pathways. KMT2A-AFF1 in silico deletion shows a general loss of stress across TFs, and may suggest TFs that have few alternative upstream regulators (Fig. 3E). Overall, this analysis shows the importance of experimental validation of the GRN, because a central node such as *MAZ* does not appear to be important for leukemia survival. However, this analysis also reveals RUNX1 to have a key role in the KMT2A-AFF1 GRN and be a promising core TF that may cooperate with KMT2A-AFF1 behavior.

### RUNX1 and KMT2A-AFF1 regulate targets in feed-forward loops and cascade motifs

**Figure 4.**
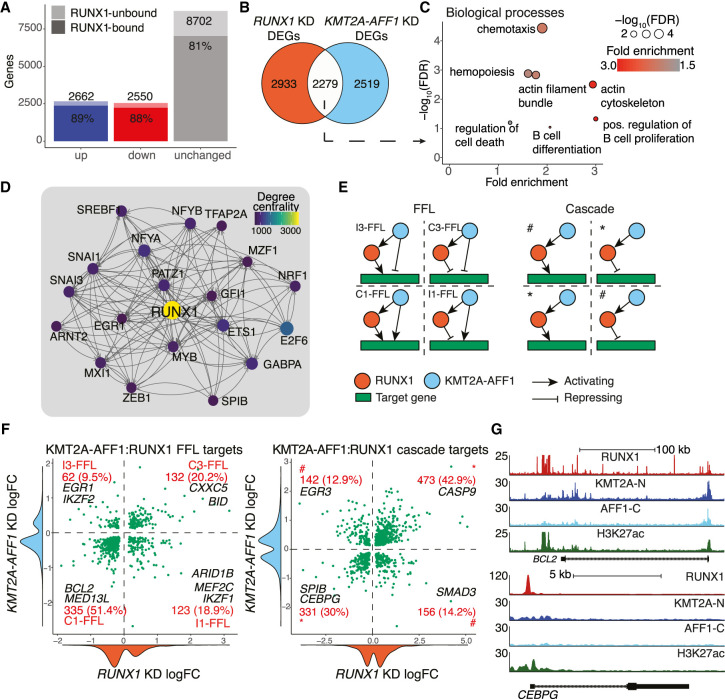
KMT2A-AFF1 cooperates with RUNX1 in FFL and cascade motifs to regulate downstream targets. (*A*) DEGs from nascent RNA-seq after 96 h *RUNX1* KD. DEGs are defined as FDR < 0.05 (*n* = 3). Shaded area represents RUNX1-bound genes. (*B*) Overlap between *KMT2A-AFF1* KD DEGs (Fig. 1C) and *RUNX1* KD DEGs (*A*). (*C*) GO biological process enrichment for overlap shown in *B*. Size of points is proportional to significance. (*D*) The top 20 genes of the RUNX1 GRN by degree centrality. Lines indicate predicted interaction from protein to gene locus, with arrowheads pointing downstream. (*E*) FFL (*left*) and cascade (*right*) motifs. FFLs are subcategorized into C1-FFL, C3-FFL, I1-FFL, and I3-FFL as indicated. Cascade motifs are grouped into same sign of effect (*) and opposing sign of effect (#). (*F*) Scatter plot of *RUNX1* and *KMT2A-AFF1* KD logFC response at FFL (*left*) and TF cascade (*right*) target genes. Density plots along the axis show distribution of logFC values. Quadrants of scatter plots align with FFL and cascade types shown in *E*. (*G*) ChIP-seq tracks for KMT2A-N, AFF1-C, RUNX1, and H3K27ac, normalized to 1 × 10^7^ reads. (*Top*) *BCL2*, a FFL target, bound by both KMT2A-AFF1 and RUNX1. (*Bottom*) *CEBPG*, a cascade target, bound by RUNX1 only.

Because RUNX1 is part of the core KMT2A-AFF1 network, we wanted to interrogate the regulatory logic of combined KMT2A-AFF1:RUNX1 activity in the GRN. Nascent RNA-seq in *RUNX1* KDs identified 5212 DEGs (Supplemental Data S3), the majority of which were bound by RUNX1 (Fig. 4A; Supplemental Fig. S6A,B). In total, 2279 genes were affected by both *RUNX1* KD and *KMT2A-AFF1* KD (Fig. 4B) and were enriched for pathways related to hematopoiesis, regulation of cell death, and B cell proliferation, consistent with a leukemic expression program (Fig. 4C; Supplemental Fig. S6C).

We created a RUNX1-centric GRN (Supplemental Data S1; Methods), and observed known targets of RUNX1 such as *GFI1* (Wilson et al. 2010) and *MYB* (Fig. 4D; Choi et al. 2017). As *RUNX1* is a direct target of KMT2A-AFF1, we expected that the RUNX1 and KMT2A-AFF1 GRNs should have commonality. We found direct KMT2A-AFF1 targets to be predominantly down-regulated with *KMT2A-AFF1* KD, *RUNX1* KD, and DOT1L inhibition, suggesting similar behavior at these loci (Supplemental Fig. S6D). *RUNX1* KD also slightly impacted KMT2A-AFF1 binding genome-wide (Supplemental Fig. S6E,F), although nascent RNA was not affected and *RUNX1* and *KMT2A-AFF1* KD logFC across DEGs does not correlate (*R* = 0.3).

To assess how RUNX1 behaves in the context of the KMT2A-AFF1 network, we integrated the two GRNs together and extracted gene targets that are regulated by network motifs involving both RUNX1 and KMT2A-AFF1 (Supplemental Data S4). Because *RUNX1* is a target of KMT2A-AFF1 (Wilkinson et al. 2013), we focused on FFLs (KMT2A-AFF1 activating RUNX1, and both KMT2A-AFF1 and RUNX1 coregulating a gene target) (Fig. 4E) and cascade motifs (KMT2A-AFF1 activating RUNX1, and RUNX1 regulating a gene target independent of KMT2A-AFF1) (Fig. 4E). FFLs can be classified as coherent (C1-FFL, C3-FFL, the same sign of effect) and incoherent (I1-FFL, I3-FFL, opposing signs), based on the regulatory effect (up- or down-regulation) on the target gene by RUNX1 and KMT2A-AFF1 (Mangan and Alon 2003).

We found 71.6% of FFLs were coherent, suggesting that KMT2A-AFF1 and RUNX1 predominantly act cooperatively (Fig. 4F, left). For example, *BCL2* (Godfrey et al. 2017) is activated in a C1-FFL (Fig. 4G; Supplemental Fig. S6G), whereas *BID* is repressed in a C3-FFL (Supplemental Fig. S6H). Similar to other analyses (Mangan et al. 2006; Joanito et al. 2018), C1-FFLs are the most common FFL in our system, which suggests that RUNX1 activity at KMT2A-AFF1 targets is biased toward gene activation.

The majority of TF cascades show agreement in transcriptional response to *KMT2A-AFF1* and *RUNX1* KD (72.9%) (Fig. 4F, right) with *CEBPG* shown as a specific example (Fig. 4G; Supplemental Fig. S6G). This is in line with our hypothesis that indirect effects of *KMT2A-AFF1* KD are mediated by TFs such as RUNX1, and suggests that RUNX1 activity is a strong determining factor for cascade motifs. Incoherent cascade motifs (27.1% of TF cascades) (Fig. 4E, denoted with #) may represent transcriptional noise or regulatory control by additional TFs. Although FFLs are activation biased, cascades show more balanced regulatory logic, suggesting that RUNX1 mediates repression as well as activation within the same regulatory network, consistent with reported RUNX1 activity in hematopoietic differentiation (Lutterbach and Hiebert 2000; Kuvardina et al. 2015).

### KMT2A-AFF1 GRN interactions are connected to venetoclax resistance and predict *CASP9* regulation through cascade motifs

Having established regulatory interplay between RUNX1 and KMT2A-AFF1, we wanted to focus on specific key genes. *BCL2* and *MYC* are key targets of KMT2A-AFF1 and promote leukemia survival (Benito et al. 2015; Godfrey et al. 2017). *KMT2A*r leukemias, as well as *MYC*-driven B cell lymphomas, are highly sensitive to inhibition of BCL2 through venetoclax treatment (Vandenberg and Cory 2013; Niu et al. 2014; Khaw et al. 2016). However venetoclax sensitivity is variable across cell line models (Pan et al. 2014), and drug resistance acquisition can be problematic. We therefore set out to ask whether any GRN motifs have the potential to mediate venetoclax resistance. For example, our GRN predicts that several TFs could regulate genes in the apoptosis pathway (Supplemental Fig. S7A).

To determine what contributes to venetoclax sensitivity and resistance, we treated an AML cell line (THP-1 cells) with venetoclax and performed a CRISPR screen (Supplemental Fig. S7B–E; Supplemental Data S5). Although SEM and THP-1 cells represent different leukemia types, the core GRN nodes are common to both AML and ALL (see Fig. 2). Because ALL leukemias have the potential to switch to AML under selective pressure, as seen with CAR-T cell therapy (Gardner et al. 2016; Pillai et al. 2019), we reasoned that important GRN motifs should be conserved. Owing to problems with maintaining library complexity in the DMSO control arm, we instead used the T0 baseline (comparing T0 with T18 venetoclax) to extract genes that mediate cell survival both generally and in the context of venetoclax. We therefore cannot distinguish whether genes are generally essential or only with venetoclax treatment. We reasoned that post-screen validations would clarify the interplay with venetoclax for key genes.

**Figure 5.**
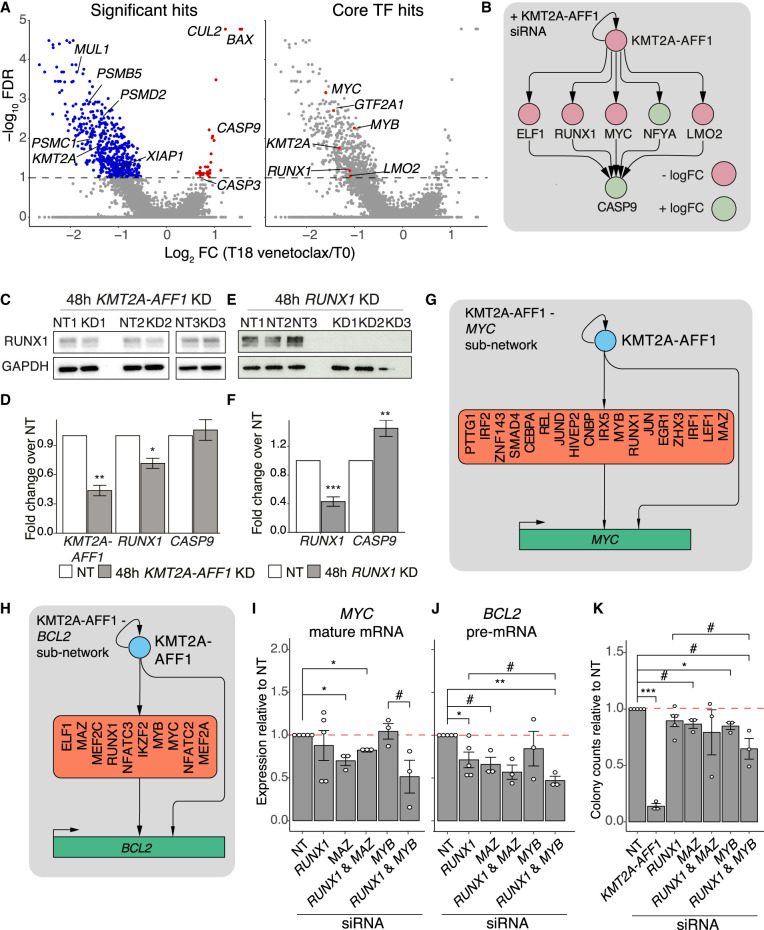
KMT2A-AFF1 and intermediate TFs cooperate to regulate cascade and FFL motif targets. (*A*) MAGeCK analysis of CRISPR screen comparing T0 (baseline) and T18 (venetoclax), plotting −log_10_ FDR against log_2_ gRNA FC. (*Left*) Key genes; (*right*) core GRN TFs. (*B*) Select cascade motifs in the KMT2A-AFF1 GRN that explain interactions between KMT2A-AFF1 and *CASP9*. Node color represents logFC response to *KMT2A-AFF1* KD. (*C*,*E*) Western blot in SEM cells showing RUNX1 protein levels after 48 h *KMT2A-AFF1* KD (*C*) or 48 h *RUNX1* KD (*E*), with GAPDH as a loading control. (*D*,*F*) qRT-PCR assaying *KMT2A-AFF1*, *RUNX1* and *CASP9* expression following 48 h *KMT2A-AFF1* KD (*D*) or 48 h *RUNX1* KD (*F*) in SEM cells (*n* = 3). Expression normalized to *GAPDH* and shown relative to NT control. (*G*,*H)* Subnetworks illustrating interactions from KMT2A-AFF1 and cooperative TFs that feed into *MYC* (*G*) and *BCL2* (*H*). (*I*,*J*) qRT-PCR analysis probing mature *MYC* mRNA (*I*) and *BCL2* pre-mRNA (*J*) after 96 h KD targeting genes as indicated (*n* = 3, *n* = 5 for NT and *RUNX1* KD). Expression normalized to *GAPDH* mature mRNA levels and shown relative to NT control. (*K*) Colony assay counts after 96 h KD targeting genes as indicated (*n* = 3, *n* = 5 for NT and *RUNX1* KD). Colony counts shown relative to NT control. Error bars represent standard error of the mean; (#) *P* < 0.1; (*) *P* < 0.05; (**) *P* < 0.01; (***) *P* < 0.001.

Depleted sgRNAs (perturbation inhibits survival) include those targeting *KMT2A*, resulting in inactivation of the essential KMT2A-MLLT3 fusion protein (Fig. 5A). Validation of this target showed that although *KMT2A* is essential for THP-1 cell survival, it does not confer significant sensitivity to venetoclax (Supplemental Fig. S7E). We also observed depleted sgRNAs for proteasome subunits (*PSMB5*, *PSMC1*, *PSMD2*), suggesting the proteasome complex promotes survival under venetoclax treatment. To validate whether essentiality is dependent on venetoclax, we used individual sgRNA to perturb several genes and confirmed that *MUL1*, *XIAP*, and *PSMD2* are essential under venetoclax conditions (Supplemental Fig. S7E).

To identify regulatory interactions that may mediate venetoclax resistance, we focused on enriched sgRNAs that are targets of KMT2A-AFF1:RUNX1 motifs. This identified *CASP9* (Fig. 5A,B), perturbation of which improves cell survival, consistent with the pro-apoptotic role of caspase 9 (Li et al. 1997; Slee et al. 1999; Li and Yuan 2008). Using individual sgRNAs, we validated that perturbation of *BAX*, *CUL2*, *CASP9*, and *CASP3* (these last two of which are in the GRN) (Supplemental Fig. S7A) confer resistance to venetoclax (Supplemental Fig. S7E). We also confirmed that *CASP9* knockouts in SEM cells reproduced the enhanced resistance to venetoclax (Supplemental Fig. S8A,B). The knockout clones showed variable RUNX1 protein and mRNA levels, but this did not correlate with *CASP9* status and is likely a result of clonal expansion (Supplemental Fig. S8C,D).

Using our GRN to predict regulation of *CASP9* we identified a repressive cascade mediated by several candidate TFs, including RUNX1 (Fig. 5B; Supplemental Fig. S8E,F). To clarify the relative contribution of KMT2A-AFF1 and RUNX1 on *CASP9* expression, we reduced the *KMT2A-AFF1* KD duration to 48 h, which reduced *RUNX1* mRNA but not protein levels (Fig. 5C). This showed that *CASP9* expression is unaffected by *KMT2A-AFF1* KD alone (*P* = 0.64) (Fig. 5D). After 48 h of *RUNX1* KD there was a loss of *RUNX1* RNA as well as protein, and *CASP9* expression increased significantly (Fig. 5E,F). This result was also reproduced in RS4;11 cells (Supplemental Fig. S8G). Together, these data validate the KMT2A-AFF1:RUNX1*:CASP9* motif and argue that KMT2A-AFF1 does not directly repress *CASP9*, but instead acts via RUNX1 (and other TFs) in a cascade (Fig. 5B).

### *BCL2* and *MYC* are regulated by multiple TFs in FFLs

In the venetoclax CRISPR screen, sgRNAs targeting core TFs of the KMT2A-AFF1 GRN, including *RUNX1*, *MYB*, and *MYC*, are depleted (Fig. 5A). This suggests that the core of the network has a role in cell survival and apoptosis. *MYC* is particularly important in relation to regulating growth and apoptosis, and overexpression is commonly paired with *BCL2* up-regulation (McMahon 2014). To better understand how core GRN TFs may contribute to cell survival, we explored the cooperative regulation of both *BCL2* and *MYC* as mediated by TFs in the GRN.

Under the KMT2A-AFF1 GRN, *BCL2* and *MYC* are regulated as FFLs controlled by KMT2A-AFF1 and several predicted TFs (Fig. 5G,H). This TF cooperation may contribute to the high expression observed in KMT2A-AFF1 leukemias (Robinson et al. 2008; Benito et al. 2015). Although perturbation of *KMT2A-AFF1* itself dysregulates *BCL2* and *MYC*, it is unclear how much of this effect is attributable to these intermediary TFs, because they are also regulated by KMT2A-AFF1. RUNX1, MAZ, and MYB are predicted to regulate both *BCL2* and *MYC* and are bound at promoters and enhancers of these loci (Supplemental Fig. S8H). We performed KDs of each TF alone or in combination with *RUNX1* (Supplemental Fig. S8I–L) and assayed *MYC* and *BCL2* expression (Fig. 5I,J). Using intronic primers to assay *BCL2* pre-mRNA (Crump et al. 2021), *RUNX1* KD alone significantly reduced *BCL2* expression, but not *MYC*. *MAZ* KD significantly reduced *MYC* expression and borderline reduced *BCL2* levels (*P* = 0.055), whereas *MYB* KD showed no effect. Combined *RUNX1* and *MYB* KD caused *MYC* and *BCL2* expression to trend toward greater down-regulation, albeit not reaching significance (*MYC P* = 0.09; *BCL2 P* = 0.063), whereas *RUNX1* and *MAZ* KD showed no additional effect. These results suggest that RUNX1 and MAZ function independently at these loci, whereas RUNX1 and MYB have some level of combinatorial regulation.

We hypothesized that this functional interaction may extend to leukemogenesis. We used colony formation assays to assess growth following combinatorial TF KD (Fig. 5K). We used a lower concentration of RUNX1 siRNA than in previous work (Wilkinson et al. 2013), so our *RUNX1* KD samples did not completely reduce *RUNX1* RNA or protein (Supplemental Fig. S8I,L), resulting in minimal and variable impact on growth (Fig. 5K). However, this reduced *RUNX1* KD allowed us to look for possible cooperative interactions with other factors. *MAZ* KD on its own slightly reduced colony forming potential (*P* = 0.075), although *RUNX1* KD did not enhance this. *MYB* KD significantly impacted growth, and when combined with *RUNX1* KD disrupted growth to a greater extent than *RUNX1* alone, although not reaching significance (*P* = 0.091). These results suggest that the combinatorial effects of RUNX1 and MYB not only regulate *MYC* and *BCL2* expression but also promote leukemogenesis. Integrating these results together, we can begin to form a picture of the upstream regulation of *MYC* and *BCL2*, and this provides a template for further studies as well as possibilities for future drug combinations.

## Discussion

*KMT2A-AFF1* KD results in both up- and down-regulation of transcription, and unexpectedly, the majority of these DEGs do not involve direct KMT2A-FP binding. This observation caused us to pose a question: If KMT2A-AFF1 predominantly functions directly through promoting transcription, by what mechanisms can it regulate gene expression in a wider network?

In this study we created a GRN that probes the relationship between KMT2A-AFF1, the primary driver of leukemic transformation (Bardini et al. 2010, 2011; Andersson et al. 2015), and the downstream transcriptional network. Our systematic approach to explain indirect KMT2A-AFF1 interactions led to the prediction of regulatory network motifs and identified a set of core factors. These core GRN factors are present in both AML and ALL leukemias and are targeted by both KMT2A-AFF1 and -MLLT3 fusion proteins. Although we focused our analysis on RUNX1, whose role in KMT2A-FP leukemias has been observed previously (Wilkinson et al. 2013; Prange et al. 2017), placing RUNX1 in the context of the KMT2A-AFF1 GRN allows us to capture complex aspects of the KMT2A-AFF1:RUNX1 regulatory axis. This is highlighted by the shared regulatory logic of RUNX1 and KMT2A-AFF1 at the majority of gene targets.

### Core network factors and motifs function as hypotheses for the upstream regulatory logic of key leukemic genes

**Figure 6.**
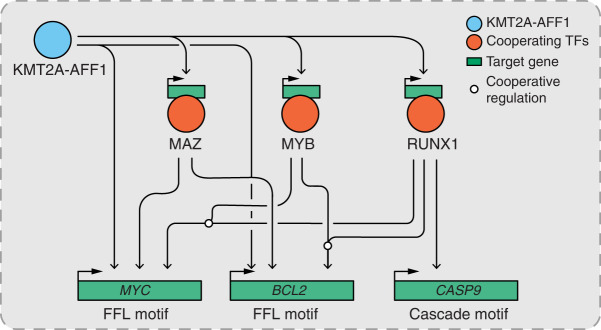
A model of KMT2A-AFF1-driven FFL and cascade network motifs that interface with *MYC*, *BCL2*, and *CASP9* genes. *MYC* and *BCL2* are regulated through many different TFs along with KMT2A-AFF1. MYB and RUNX1 act in combination at these loci, where combined disruption appears to enhance *MYC* dysregulation. *CASP9* is regulated in a cascade motif, with RUNX1 acting as an intermediate node, where disruption induces *CASP9* up-regulation.

We used this GRN to make predictions about how cell behavior is regulated through KMT2A-AFF1:TF driven motifs. We initially asked how these motifs may associate with venetoclax resistance mechanisms, and in doing so highlighted a number of core TFs that impact cell survival. How these factors mediate venetoclax sensitivity and cell survival is likely to be multifaceted, but we explored key pathways that influence apoptosis or growth. We identified a KMT2A-AFF1:RUNX1:*CASP9* cascade, where RUNX1 acts as an intermediate repressor of *CASP9*. Although *CASP9* is mostly regulated on the protein level, subtle impacts on the expression of the *CASP9* gene could influence leukemia survival over the longer term. Other key factors important for leukemia survival include *MYC* and *BCL2*, particularly in relation to venetoclax treatment. Our analyses identify upstream combinatorial regulation of these genes via FFL motifs, where the core TFs RUNX1 and MYB appear to synergistically regulate these and likely other key gene targets (Fig. 6). Our data implies an interplay between different types of regulatory motifs, because the KMT2A-AFF1:TF:*CASP9* repressive cascades identified here may cooperate with FFLs activating *BCL2* and *MYC* to prevent cell death (Fig. 6). This provides a potentially useful paradigm in which simple patterns of cascade and FFL motifs can cooperate in regulating complex pathways.

### Regulatory interactions that describe the GRN model may function as the basis for understanding leukemic behavior

A concept briefly discussed in a review from the Pimanda laboratory (Thoms et al. 2019) is that AML is caused by dysregulation of an entire transcriptional network. This would explain why we observe such a wide range of chromatin/transcription-associated driver mutations that may function through disruption of the same transcriptional motifs. Our study reinforces this concept as we see that the core GRN is expressed across different ALL and AML patients, which we consider core TFs attributable to general KMT2A-FP behavior. This may also help to explain why KMT2A-AFF1 is sufficient to drive leukemogenesis in the absence of other cooperating mutations, because these core factors impact a range of biological systems. The development of a cancer cell requires the disruption of multiple processes, usually necessitating multiple mutations (Hanahan and Weinberg 2011). However, this core KMT2A-AFF1 network may be able to co-opt multiple pathways to achieve a similar result. Considering *KMT2A*r ALLs are known to switch to an AML lineage (Dorantes-Acosta and Pelayo 2012; Gardner et al. 2016), one would expect these core factors to be conserved between these leukemias, because they could represent common regulatory wiring that enables an oncogenic transcription program. Because the core of the GRN is active across multiple leukemia data sets, this further implies that our core *KMT2A*r driven TFs attribute to multiple types of leukemia.

## Methods

### Cell line culture

SEM cells, a KMT2A-AFF1 B cell ALL line (Greil et al. 1994), were purchased from DSMZ (https://www.dsmz.de). SEM cells were cultured in Iscove's modified Dulbecco's medium (IMDM) with 10% fetal bovine serum (FBS) and 1× GlutaMAX, with cell density maintained between 5 × 10^5^/mL and 2 × 10^6^/mL. RS4;11 and THP-1 cells were purchased from ATCC (https://www.atcc.org). RS4;11 and THP-1 cells were cultured in RPMI-1640 with 10% FBS and 1× GlutaMAX, with cell density maintained between 5 × 10^5^/mL and 1.5 × 10^6^/mL. Cells were confirmed to be free of mycoplasma.

### Patient samples

KMT2A-AFF1 patient sample 1 is described in Kerry et al. (2017). KMT2A-AFF1 patient sample 2 is a primary diagnostic bone marrow sample from a 6-yr-old child with ALL, obtained from the Bloodwise Childhood Leukaemia Cell Bank, UK (REC: 16/SW/0219). Samples were anonymized at source, assigned a unique study number, and linked.

### siRNA knockdowns

siRNA KDs were performed as previously described (Kerry et al. 2017). For 96 h KD cells were retransfected 48 h after initial transfection. The following siRNA were used: SEM *KMT2A-AFF1* siRNA (siMA6), RS4;11 *KMT2A-AFF1* siRNA (siMARS), and scrambled control (siMM) (Thomas et al. 2005); *RUNX1* (Dharmacon ON-TARGETplus single siRNA, J-003926-05); *MAZ* (Ambion Silencer Select, s8543); *MYB* (Ambion Silencer Select, s9110); non-targeting controls (Dharmacon ON-TARGETplus non-targeting pool, D-001810-10-20).

### Western blots

Proteins were extracted using BC300 lysis buffer with protease inhibitors. Western blotting was performed as previously described (Wilkinson et al. 2013). Antibodies used were all raised in rabbit and are detailed in Supplemental Table S1.

### qRT-PCR

Total RNA was extracted using the RNeasy Mini kit (Qiagen) after 48 or 96 h KD. cDNA was generated using SuperScript III Reverse Transcriptase (Invitrogen) with random hexamer primers. qRT-PCR analysis was performed using TaqMan or SYBR probes and analyzed with the ΔΔCt method normalizing to the housekeeping gene *GAPDH*. Primers used are detailed in Supplemental Table S2. Statistical analyses were performed using two-sided student's *t*-test.

### Nascent RNA-seq

Nascent RNA-seq was performed as previously described (Kerry et al. 2017), after 96 h *RUNX1* (SEM) or *KMT2A-AFF1* (RS4;11) KD. Briefly, 1 × 10^8^ SEM cells were treated with 500 μM 4-thiouridine (4-SU) for 1 h. Cells were lysed with TRIzol (Invitrogen), and total RNA was precipitated and DNase I-treated. 4-SU-incorporated RNA was purified by biotinylation and streptavidin bead pulldown. DNA libraries were prepared using the Ultra II Directional RNA library prep kit (NEB, E7765) and sequenced by paired-end sequencing using a NextSeq 500 (Illumina).

### RNA-seq analysis

FASTQ files were quality checked using FastQC (v0.11.4) and reads trimmed using Trim Galore! (v0.4.1) (Martin 2011). Paired-end reads were mapped to hg19 using STAR (v2.4.2) (Dobin et al. 2013). We mapped to hg19 for compatibility with previously published data sets. Blacklisted regions were removed, and uniquely mapped reads were used for processing. Therefore, our choice of hg19 over GRCh38 should not significantly affect results. PCR duplicates were removed using Picard-tools MarkDuplicates (v1.83). Mapped reads over exons were quantified using subread featureCounts (v1.6.2) (Liao et al. 2014). Statistical analysis was performed in R (R Core Team 2021) using the edgeR package (Robinson et al. 2010). DEGs were defined as FDR < 0.05. Enriched GO terms and reactome pathways were determined using PANTHER (v. 15) (Thomas et al. 2003). Published expression data was sourced from NCBI Gene Expression Omnibus (GEO; https://www.ncbi.nlm.nih.gov/geo/) accessions GSE85988, GSE83671, and GSE139437 (Supplemental Table S3).

### Chromatin Immunoprecipitation (ChIP)

The cells (1 × 10^7–8^) were fixed and sonicated using a Covaris ME220 according to the manufacturer's recommendations. Ab:chromatin complexes were isolated using magnetic Protein A and Protein G Dynabeads (Invitrogen) and washed three times with 50 mM HEPES-KOH, pH 7.6, 500 mM LiCl, 1 mM EDTA, 1% NP-40, and 0.7% Na deoxycholate. Antibodies used are in Supplemental Table S1. Samples were washed with Tris-EDTA, eluted, and treated with RNase A and Proteinase K. DNA was purified with a Qiagen PCR purification kit. Samples were analyzed by ChIP qPCR or ChIP-sequencing. Reference normalization (Orlando et al. 2014) was performed by adding fixed *Drosophila melanogaster* S2 cells before sonication in a 1:5 ratio. Primers used for qPCR analysis are listed in Supplemental Table S2. Libraries were generated using Ultra II DNA library preparation kit for Illumina (NEB) and sequenced using a NextSeq 500 (Illumina).

### ChIP-seq analysis

Alignment, PCR duplicate filtering, and blacklisted region filtering was performed using the NGseqBasic pipeline (Telenius et al. 2018). Briefly, FASTQ files were quality checked using FastQC (v0.11.4) and mapped using Bowtie (v1.0.0) (Langmead 2010) against hg19. Unmapped reads were trimmed with Trim Galore! (v0.3.1) (Martin 2011) and remapped. Short unmapped reads were combined using FLASH (v1.2.8) (Magoč and Salzberg 2011) and remapped. PCR duplicates were removed using SAMtools rmdup (v0.1.19) (Li et al. 2009). Reads mapping to Duke blacklisted regions (UCSC) were removed using BEDtools (v2.17.0) (Quinlan and Hall 2010). We used hg19 to preserve compatibility with older data sets. We tested whether peak-promoter annotations would be significantly changed using GRCh38 and found >99% of annotations are preserved. For reference normalization, input and IP reads were mapped to hg19 and dm3 genome builds, and hg19 read counts adjusted based on the ratio of dm3:hg19 reads in input and IP control/treatment samples. ChIP-seq tag (read) directories and bigWigs were made using HOMER (v4.7) (Heinz et al. 2010), normalizing tag counts to tags per 10 million tags. KMT2A-N and AFF1-C peaks were called using SeqMonk (v0.24.1), and TF peaks were called using HOMER findPeaks (-style factor), with input tracks for background correction. KMT2A-AFF1 peaks were defined as overlapping KMT2A-N and AFF1-C peaks, with at least a 1-bp overlay. Peaks were annotated to the nearest promoter using HOMER annotatePeaks.pl (v4.8). Heatmaps and scatter plots were made using deepTools2 (v3.0.1) (Ramírez et al. 2016). Published data were sourced from NCBI GEO accessions GSE74812, GSE42075, GSE117865, and GSE83671 (Supplemental Table S3).

### GRN creation and analysis

GRNs were created on the basis of a central node: KMT2A-AFF1 or RUNX1. Construction involved four steps (Fig. 1D). (1) DEGs from nascent RNA-seq data after 96 h *KMT2A-AFF1* KD (Kerry et al. 2017), 96 h *RUNX1* KD, 7 d EPZ-5676 (DOT1L inhibition) (Godfrey et al. 2019), or 1.5 h IBET-151 (BRD4 inhibition) (Crump et al. 2021) were considered the regulatory scope. (2) Direct interactions were established using ChIP-seq data for the corresponding central node (H3K79me3 for DOT1L) with promoter-peak annotations. Only RUNX1 peaks overlapping enhancers (H3K27ac overlapped with H3K4me1) were used. At least 1-bp overlap was considered sufficient to filter out nonregulatory RUNX1 binding and avoid erroneous removal of regulatory binding. (3) Indirect interactions were integrated with the R package GeneNetworkBuilder (v1.26.1) using TF interaction data from the FANTOM consortium (The FANTOM Consortium and the Riken Omics Science Center 2009). TF interactions that do not explain connectivity between the central node and downstream targets were excluded. (4) Nodes of the network were annotated using ALL and AML patient RNA-seq (Methods) and CRISPR screens (Tzelepis et al. 2016; Behan et al. 2019). Degree and stress centralities were calculated using the R packages igraph (v1.2.4.1) and sna (v2.4).

### Patient subnetwork creation and analysis

We used published RNA-seq data to create subgraphs of the KMT2A-AFF1 GRN. Data include *KMT2A*r ALL patients (raw data available at NCBI BioProject database; https://www.ncbi.nlm.nih.gov/bioproject/ under accession number PRJEB23605) (Agraz-Doblas et al. 2019), AML patients with a range of chromosomal abnormalities (expression tables available from Genomic Data Commons [GDC] of the National Cancer Institute; https://gdc.cancer.gov/about-data/publications/laml_2012) (The Cancer Genome Atlas Research Network 2013), and normal FBM (*n* = 3 samples; raw data available at NCBI GEO accession GSE122982) (O'Byrne et al. 2019). Subnetworks were derived by filtering the *KMT2A-AFF1* KD RNA-seq for genes expressed in each RNA-seq sample and reprocessing the GRN workflow. Expressed genes are defined as greater than mean log_2_ TPM, calculated for each data set separately (Supplemental Fig. S3A). Node presence across all subnetworks were converted into a binary matrix, and UMAP dimensionality and *k*-means clustering was performed using R (R Core Team 2021).

### Annexin V/PI assay

Annexin V/PI assay was used to determine viability after venetoclax treatment, as described previously (Souers et al. 2013; Pan et al. 2014). THP-1 cells were treated for 48 h with a range of venetoclax concentrations. Cells were incubated in 50 μL annexin V binding buffer with FITC-conjugated annexin V (BioLegend) and propidium iodide (PI, ChemoMetec) for 15 min on ice. Stained cells were analyzed using an Attune NxT flow cytometer (Thermo Fisher Scientific).

### CRISPR screen generation

Lentiviral CRISPR knockout screening was conducted with the TKOv3 genome-wide sgRNA library as described previously (Aregger et al. 2019) with the following modifications. HEK293T cells were transfected with pooled library plasmid, pMD2.G, and psPAX2 plasmids using PEI Pro (Polyplus Transfection). Harvests were collected 48- and 72-h post transfection, and viral supernatants filtered through a 0.45 µM cellulose acetate filter. The single-vector TKOv3-lentiCRISPRv2 library was a gift from Jason Moffat (Addgene 90294) (Hart et al. 2017). Library representation was maintained at a minimum of 250 cells/sgRNA. The screen was performed in duplicate with THP-1 cells transduced with TKOv3 lentivirus at MOI = 0.3 (T-6). After 72 h, transduced cells were selected with 2 μg/mL puromycin (T-3). After 72 h selection, 1.8 × 10^7^ viable cells were harvested (T0). Cells were cultured with 7.4 μM venetoclax (ABT-199, Stratech. IC_50_ in THP-1 cells) (Supplemental Fig. S7C) for 18 d (T18, six passages), and harvested. gDNA was isolated with QIAamp DNA Blood Maxi Kit (Qiagen), and sgRNA libraries were generated using two-step PCR and sequenced by paired-end sequencing using a NextSeq 500 (Illumina).

### CRISPR screen analysis

sgRNA reads were merged using BBMerge and aligned to the TKOv3 library using CRISPressoCount (Canver et al. 2018) with a quality score threshold of 30. sgRNA counts were analyzed with MAGeCK-RRA (v0.5.7) (Li et al. 2014) using copy number variation (CNV) data for THP-1 cells from the Cancer Cell Line Encyclopedia as previously described (Meyers et al. 2017). Robust rank aggregation was performed with 10,000 permutations.

### CellTiter-Glo for screen validations

To validate CRISPR screen hits, individual sgRNA sequences were cloned into lentiCRISPRv2 (gifted by Feng Zhang) (Addgene 52961) and used to target SEM or THP-1 cells. CRISPR-Cas9 edited SEM or THP-1 cells were treated for 48 h with 2.5 μM or 20 μM venetoclax, respectively. Viability was assayed using CellTiter-Glo (CTG) luminescence assay. Cell suspensions were mixed at a 1:1 ratio with CTG substrate, and luminescence was detected using a BMG FLUOstar OPTIMA plate reader. Viability for THP-1 cells were shown relative to *AAVS1* targeting sgRNA (Sadelain et al. 2012) and statistical analysis was performed using two-sided student's *t*-test.

### Colony-forming assay

Cells were transfected with siRNA, and 48 h later retransfected with the same siRNA. Then 24 h later 500 cells were plated in IMDM MethoCult media with 20% FCS (H4100 STEMCELL technologies) per dish, in triplicate. Colonies were incubated for 14 d (37°C, 5% CO_2_) before counting.

## Data access

Previously published sequencing data are available from GEO accession numbers reported in Methods and Supplemental Table S3. All raw and processed sequencing data generated in this study have been submitted to the NCBI Gene Expression Omnibus (GEO; https://www.ncbi.nlm.nih.gov/geo/) under accession number GSE151390. Custom R scripts used in this study are available as Supplemental Code and at GitHub (https://github.com/JoeHarman/MLLAF4-GRN_paper_2021).

## Supplementary Material

Supplemental Material

## References

[GR268490HARC1] Agraz-Doblas A, Bueno C, Bashford-Rogers R, Roy A, Schneider P, Bardini M, Ballerini P, Cazzaniga G, Moreno T, Revilla C, 2019. Unraveling the cellular origin and clinical prognostic markers of infant B-cell acute lymphoblastic leukemia using genome-wide analysis. Haematologica 104: 1176–1188. 10.3324/haematol.2018.206375 30679323 PMC6545849

[GR268490HARC2] Aittokallio T, Schwikowski B. 2006. Graph-based methods for analysing networks in cell biology. Brief Bioinform 7: 243–255. 10.1093/bib/bbl022 16880171

[GR268490HARC3] Alexander TB, Gu Z, Iacobucci I, Dickerson K, Choi JK, Xu B, Payne-Turner D, Yoshihara H, Loh ML, Horan J, 2018. The genetic basis and cell of origin of mixed phenotype acute leukaemia. Nature 562: 373–379. 10.1038/s41586-018-0436-0 30209392 PMC6195459

[GR268490HARC4] Andersson AK, Ma J, Wang J, Chen X, Gedman AL, Dang J, Nakitandwe J, Holmfeldt L, Parker M, Easton J, 2015. The landscape of somatic mutations in infant MLL-rearranged acute lymphoblastic leukemias. Nat Genet 47: 330–337. 10.1038/ng.3230 25730765 PMC4553269

[GR268490HARC5] Aregger M, Chandrashekhar M, Tong AHY, Chan K, Moffat J. 2019. Pooled lentiviral CRISPR-Cas9 screens for functional genomics in mammalian cells. Methods Mol Biol 1869: 169–188. 10.1007/978-1-4939-8805-1_15 30324523

[GR268490HARC6] Assi SA, Imperato MR, Coleman DJL, Pickin A, Potluri S, Ptasinska A, Chin PS, Blair H, Cauchy P, James SR, 2019. Subtype-specific regulatory network rewiring in acute myeloid leukemia. Nat Genet 51: 151–162. 10.1038/s41588-018-0270-1 30420649 PMC6330064

[GR268490HARC7] Ballabio E, Milne TA. 2012. Molecular and epigenetic mechanisms of MLL in human leukemogenesis. Cancers (Basel) 4: 904–944. 10.3390/cancers4030904 24213472 PMC3712720

[GR268490HARC8] Bardini M, Spinelli R, Bungaro S, Mangano E, Corral L, Cifola I, Fazio G, Giordan M, Basso G, De Rossi G, 2010. DNA copy-number abnormalities do not occur in infant ALL with t(4;11)/*MLL-AF4*. Leukemia 24: 169–176. 10.1038/leu.2009.203 19907438

[GR268490HARC9] Bardini M, Galbiati M, Lettieri A, Bungaro S, Gorletta TA, Biondi A, Cazzaniga G. 2011. Implementation of array based whole-genome high-resolution technologies confirms the absence of secondary copy-number alterations in *MLL-AF4*-positive infant ALL patients. Leukemia 25: 175–178. 10.1038/leu.2010.232 20944671

[GR268490HARC10] Baxter E, Windloch K, Gannon F, Lee JS. 2014. Epigenetic regulation in cancer progression. Cell Biosci 4: 45. 10.1186/2045-3701-4-45 25949794 PMC4422217

[GR268490HARC11] Behan FM, Iorio F, Picco G, Gonçalves E, Beaver CM, Migliardi G, Santos R, Rao Y, Sassi F, Pinnelli M, 2019. Prioritization of cancer therapeutic targets using CRISPR–Cas9 screens. Nature 568: 511–516. 10.1038/s41586-019-1103-9 30971826

[GR268490HARC12] Benito JM, Godfrey L, Kojima K, Hogdal L, Wunderlich M, Geng H, Marzo I, Harutyunyan KG, Golfman L, North P, 2015. MLL-rearranged acute lymphoblastic leukemias activate BCL-2 through H3K79 methylation and are sensitive to the BCL-2-specific antagonist ABT-199. Cell Rep 13: 2715–2727. 10.1016/j.celrep.2015.12.003 26711339 PMC4700051

[GR268490HARC13] Bernt KM, Zhu N, Sinha AU, Vempati S, Faber J, Krivtsov AV, Feng Z, Punt N, Daigle A, Bullinger L, 2011. *MLL*-rearranged leukemia is dependent on aberrant H3K79 methylation by DOT1L. Cancer Cell 20: 66–78. 10.1016/j.ccr.2011.06.010 21741597 PMC3329803

[GR268490HARC14] Bhattacharjee S, Renganaath K, Mehrotra R, Mehrotra S. 2013. Combinatorial control of gene expression. Biomed Res Int 2013: 407263. 10.1155/2013/407263 24069600 PMC3771257

[GR268490HARC15] Biswas D, Milne TA, Basrur V, Kim J, Elenitoba-Johnson KSJ, Allis CD, Roeder RG. 2011. Function of leukemogenic mixed lineage leukemia 1 (MLL) fusion proteins through distinct partner protein complexes. Proc Natl Acad Sci 108: 15751–15756. 10.1073/pnas.1111498108 21896721 PMC3179097

[GR268490HARC16] The Cancer Genome Atlas Research Network. 2013. Genomic and epigenomic landscapes of adult de novo acute myeloid leukemia. N Engl J Med 368: 2059–2074. 10.1056/NEJMoa1301689 23634996 PMC3767041

[GR268490HARC17] Canver MC, Haeussler M, Bauer DE, Orkin SH, Sanjana NE, Shalem O, Yuan GC, Zhang F, Concordet JP, Pinello L. 2018. Integrated design, execution, and analysis of arrayed and pooled CRISPR genome-editing experiments. Nat Protoc 13: 946–986. 10.1038/nprot.2018.005 29651054 PMC6182299

[GR268490HARC18] Choi A, Illendula A, Pulikkan JA, Roderick JE, Tesell J, Yu J, Hermance N, Zhu LJ, Castilla LH, Bushweller JH, 2017. RUNX1 is required for oncogenic *Myb* and *Myc* enhancer activity in T-cell acute lymphoblastic leukemia. Blood 130: 1722–1733. 10.1182/blood-2017-03-775536 28790107 PMC5639483

[GR268490HARC19] Crump NT, Ballabio E, Godfrey L, Thorne R, Repapi E, Kerry J, Tapia M, Hua P, Lagerholm C, Filippakopoulos P, 2021. BET inhibition disrupts transcription but retains enhancer-promoter contact. Nat Commun 12: 223. 10.1038/s41467-020-20400-z 33431820 PMC7801379

[GR268490HARC20] Dawson MA, Prinjha RK, Dittmann A, Giotopoulos G, Bantscheff M, Chan WI, Robson SC, Chung C, Hopf C, Savitski MM, 2011. Inhibition of BET recruitment to chromatin as an effective treatment for MLL-fusion leukaemia. Nature 478: 529–533. 10.1038/nature10509 21964340 PMC3679520

[GR268490HARC21] de Matos Simoes R, Dehmer M, Emmert-Streib F. 2013. B-cell lymphoma gene regulatory networks: biological consistency among inference methods. Front Genet 4: 281. 10.3389/fgene.2013.00281 24379827 PMC3864360

[GR268490HARC22] Dobin A, Davis CA, Schlesinger F, Drenkow J, Zaleski C, Jha S, Batut P, Chaisson M, Gingeras TR. 2013. STAR: ultrafast universal RNA-seq aligner. Bioinformatics 29: 15–21. 10.1093/bioinformatics/bts635 23104886 PMC3530905

[GR268490HARC23] Doench JG, Fusi N, Sullender M, Hegde M, Vaimberg EW, Donovan KF, Smith I, Tothova Z, Wilen C, Orchard R, 2016. Optimized sgRNA design to maximize activity and minimize off-target effects of CRISPR-Cas9. Nat Biotechnol 34: 184–191. 10.1038/nbt.3437 26780180 PMC4744125

[GR268490HARC24] Dorantes-Acosta E, Pelayo R. 2012. Lineage switching in acute leukemias: a consequence of stem cell plasticity? Bone Marrow Res 2012: 406796. 10.1155/2012/406796 22852088 PMC3407598

[GR268490HARC25] The FANTOM Consortium and the Riken Omics Science Center. 2009. The transcriptional network that controls growth arrest and differentiation in a human myeloid leukemia cell line. Nat Genet 41: 553–562. 10.1038/ng.375 19377474 PMC6711855

[GR268490HARC26] Gardner R, Wu D, Cherian S, Fang M, Hanafi LA, Finney O, Smithers H, Jensen MC, Riddell SR, Maloney DG, 2016. Acquisition of a CD19-negative myeloid phenotype allows immune escape of *MLL*-rearranged B-ALL from CD19 CAR-T-cell therapy. Blood 127: 2406–2410. 10.1182/blood-2015-08-665547 26907630 PMC4874221

[GR268490HARC27] Geng H, Brennan S, Milne TA, Chen WY, Li Y, Hurtz C, Kweon SM, Zickl L, Shojaee S, Neuberg D, 2012. Integrative epigenomic analysis identifies biomarkers and therapeutic targets in adult B-acute lymphoblastic leukemia. Cancer Discov 2: 1004–1023. 10.1158/2159-8290.CD-12-0208 23107779 PMC3516186

[GR268490HARC28] Georgescu C, Longabaugh WJR, Scripture-Adams DD, David-Fung ES, Yui MA, Zarnegar MA, Bolouri H, Rothenberg EV. 2008. A gene regulatory network armature for T lymphocyte specification. Proc Natl Acad Sci 105: 20100–20105. 10.1073/pnas.0806501105 19104054 PMC2629331

[GR268490HARC29] Ghasemi M, Seidkhani H, Tamimi F, Rahgozar M, Masoudi-Nejad A. 2014. Centrality measures in biological networks. Curr Bioinform 9: 426–441. 10.2174/15748936113086660013

[GR268490HARC30] Godfrey L, Kerry J, Thorne R, Repapi E, Davies JOJ, Tapia M, Ballabio E, Hughes JR, Geng H, Konopleva M, 2017. MLL-AF4 binds directly to a BCL-2 specific enhancer and modulates H3K27 acetylation. Exp Hematol 47: 64–75. 10.1016/j.exphem.2016.11.003 27856324 PMC5333536

[GR268490HARC31] Godfrey L, Crump NT, Thorne R, Lau IJ, Repapi E, Dimou D, Smith AL, Harman JR, Telenius JM, Oudelaar AM, 2019. DOT1L inhibition reveals a distinct subset of enhancers dependent on H3K79 methylation. Nat Commun 10: 2803. 10.1038/s41467-019-10844-3 31243293 PMC6594956

[GR268490HARC32] Goode DK, Obier N, Vijayabaskar MS, Lie-A-Ling M, Lilly AJ, Hannah R, Lichtinger M, Batta K, Florkowska M, Patel R, 2016. Dynamic gene regulatory networks drive hematopoietic specification and differentiation. Dev Cell 36: 572–587. 10.1016/j.devcel.2016.01.024 26923725 PMC4780867

[GR268490HARC33] Greil J, Gramatzki M, Burger R, Marschalek R, Peltner M, Trautmann U, Hansen-Hagge TE, Bartram CR, Fey GH, Stehr K. 1994. The acute lymphoblastic leukaemia cell line SEM with t(4;11) chromosomal rearrangement is biphenotypic and responsive to interleukin-7. Br J Haematol 86: 275–283. 10.1111/j.1365-2141.1994.tb04726.x 8199015

[GR268490HARC34] Guenther MG, Lawton LN, Rozovskaia T, Frampton GM, Levine SS, Volkert TL, Croce CM, Nakamura T, Canaani E, Young RA. 2008. Aberrant chromatin at genes encoding stem cell regulators in human mixed-lineage leukemia. Genes Dev 22: 3403–3408. 10.1101/gad.1741408 19141473 PMC2607073

[GR268490HARC35] Hanahan D, Weinberg RA. 2011. Hallmarks of cancer: the next generation. Cell 144: 646–674. 10.1016/j.cell.2011.02.013 21376230

[GR268490HARC36] Hart T, Tong AHY, Chan K, Van Leeuwen J, Seetharaman A, Aregger M, Chandrashekhar M, Hustedt N, Seth S, Noonan A, 2017. Evaluation and design of genome-wide CRISPR/SpCas9 knockout screens. G3 (Bethesda) 7: 2719–2727. 10.1534/g3.117.041277 28655737 PMC5555476

[GR268490HARC37] Heinz S, Benner C, Spann N, Bertolino E, Lin YC, Laslo P, Cheng JX, Murre C, Singh H, Glass CK. 2010. Simple combinations of lineage-determining transcription factors prime *cis*-regulatory elements required for macrophage and B cell identities. Mol Cell 38: 576–589. 10.1016/j.molcel.2010.05.004 20513432 PMC2898526

[GR268490HARC38] Ingram PJ, Stumpf MP, Stark J. 2006. Network motifs: structure does not determine function. BMC Genomics 7: 108. 10.1186/1471-2164-7-108 16677373 PMC1488845

[GR268490HARC39] Joanito I, Chu JW, Wu SH, Hsu CP. 2018. An incoherent feed-forward loop switches the *Arabidopsis* clock rapidly between two hysteretic states. Sci Rep 8: 13944. 10.1038/s41598-018-32030-z 30224713 PMC6141573

[GR268490HARC40] Kerry J, Godfrey L, Repapi E, Tapia M, Blackledge NP, Ma H, Ballabio E, O'Byrne S, Ponthan F, Heidenreich O, 2017. MLL-AF4 spreading identifies binding sites that are distinct from super-enhancers and that govern sensitivity to DOT1L inhibition in leukemia. Cell Rep 18: 482–495. 10.1016/j.celrep.2016.12.054 28076791 PMC5263239

[GR268490HARC41] Khaw SL, Suryani S, Evans K, Richmond J, Robbins A, Kurmasheva RT, Billups CA, Erickson SW, Guo Y, Houghton PJ, 2016. Venetoclax responses of pediatric ALL xenografts reveal sensitivity of MLL-rearranged leukemia. Blood 128: 1382–1395. 10.1182/blood-2016-03-707414 27343252 PMC5016707

[GR268490HARC42] Krivtsov AV, Armstrong SA. 2007. MLL translocations, histone modifications and leukaemia stem-cell development. Nat Rev Cancer 7: 823–833. 10.1038/nrc2253 17957188

[GR268490HARC43] Krivtsov AV, Feng Z, Lemieux ME, Faber J, Vempati S, Sinha AU, Xia X, Jesneck J, Bracken AP, Silverman LB, 2008. H3K79 methylation profiles define murine and human MLL-AF4 leukemias. Cancer Cell 14: 355–368. 10.1016/j.ccr.2008.10.001 18977325 PMC2591932

[GR268490HARC44] Kuvardina ON, Herglotz J, Kolodziej S, Kohrs N, Herkt S, Wojcik B, Oellerich T, Corso J, Behrens K, Kumar A, 2015. RUNX1 represses the erythroid gene expression program during megakaryocytic differentiation. Blood 125: 3570–3579. 10.1182/blood-2014-11-610519 25911237 PMC4463808

[GR268490HARC45] Lacoste N, Utley RT, Hunter JM, Poirier GG, Côte J. 2002. Disruptor of telomeric silencing-1 is a chromatin-specific histone H3 methyltransferase. J Biol Chem 277: 30421–30424. 10.1074/jbc.C200366200 12097318

[GR268490HARC46] Landt SG, Marinov GK, Kundaje A, Kheradpour P, Pauli F, Batzoglou S, Bernstein BE, Bickel P, Brown JB, Cayting P, 2012. ChIP-seq guidelines and practices of the ENCODE and modENCODE Consortia. Genome Res 22: 1813–1831. 10.1101/gr.136184.111 22955991 PMC3431496

[GR268490HARC47] Langmead B. 2010. Aligning short sequencing reads with Bowtie. Curr Protoc Bioinformatics CHAPTER 11: Unit-11.7. 10.1002/0471250953.bi1107s32 PMC3010897 21154709

[GR268490HARC48] Lee TI, Rinaldi NJ, Robert F, Odom DT, Bar-Joseph Z, Gerber GK, Hannett NM, Harbison CT, Thompson CM, Simon I, 2002. Transcriptional regulatory networks in *Saccharomyces cerevisiae*. Science 298: 799–804. 10.1126/science.1075090 12399584

[GR268490HARC49] Li J, Yuan J. 2008. Caspases in apoptosis and beyond. Oncogene 27: 6194–6206. 10.1038/onc.2008.297 18931687

[GR268490HARC50] Li P, Nijhawan D, Budihardjo I, Srinivasula SM, Ahmad M, Alnemri ES, Wang X. 1997. Cytochrome c and dATP-dependent formation of Apaf-1/caspase-9 complex initiates an apoptotic protease cascade. Cell 91: 479–489. 10.1016/S0092-8674(00)80434-1 9390557

[GR268490HARC51] Li H, Handsaker B, Wysoker A, Fennell T, Ruan J, Homer N, Marth G, Abecasis G, Durbin R, 1000 Genome Project Data Processing Subgroup. 2009. The Sequence Alignment/Map format and SAMtools. Bioinformatics 25: 2078–2079. 10.1093/bioinformatics/btp352 19505943 PMC2723002

[GR268490HARC52] Li W, Xu H, Xiao T, Cong L, Love MI, Zhang F, Irizarry RA, Liu JS, Brown M, Liu XS. 2014. MAGeCK enables robust identification of essential genes from genome-scale CRISPR/Cas9 knockout screens. Genome Biol 15: 554. 10.1186/s13059-014-0554-4 25476604 PMC4290824

[GR268490HARC53] Liao Y, Smyth GK, Shi W. 2014. featureCounts: an efficient general purpose program for assigning sequence reads to genomic features. Bioinformatics 30: 923–930. 10.1093/bioinformatics/btt656 24227677

[GR268490HARC54] Lin C, Smith ER, Takahashi H, Lai KC, Martin-Brown S, Florens L, Washburn MP, Conaway JW, Conaway RC, Shilatifard A. 2010. AFF4, a component of the ELL/P-TEFb elongation complex and a shared subunit of MLL chimeras, can link transcription elongation to leukemia. Mol Cell 37: 429–437. 10.1016/j.molcel.2010.01.026 20159561 PMC2872029

[GR268490HARC55] Lin S, Luo RT, Ptasinska A, Kerry J, Assi SA, Wunderlich M, Imamura T, Kaberlein JJ, Rayes A, Althoff MJ, 2016. Instructive role of MLL-fusion proteins revealed by a model of t(4;11) Pro-B acute lymphoblastic leukemia. Cancer Cell 30: 737–749. 10.1016/j.ccell.2016.10.008 27846391

[GR268490HARC56] Lopes-Ramos CM, Paulson JN, Chen CY, Kuijjer ML, Fagny M, Platig J, Sonawane AR, DeMeo DL, Quackenbush J, Glass K. 2017. Regulatory network changes between cell lines and their tissues of origin. BMC Genomics 18: 723. 10.1186/s12864-017-4111-x 28899340 PMC5596945

[GR268490HARC57] Lutterbach B, Hiebert SW. 2000. Role of the transcription factor AML-1 in acute leukemia and hematopoietic differentiation. Gene 245: 223–235. 10.1016/S0378-1119(00)00014-7 10717473

[GR268490HARC58] Magoč T, Salzberg SL. 2011. FLASH: fast length adjustment of short reads to improve genome assemblies. Bioinformatics 27: 2957–2963. 10.1093/bioinformatics/btr507 21903629 PMC3198573

[GR268490HARC59] Mangan S, Alon U. 2003. Structure and function of the feed-forward loop network motif. Proc Natl Acad Sci 100: 11980–11985. 10.1073/pnas.2133841100 14530388 PMC218699

[GR268490HARC60] Mangan S, Itzkovitz S, Zaslaver A, Alon U. 2006. The incoherent feed-forward loop accelerates the response-time of the gal system of *Escherichia coli*. J Mol Biol 356: 1073–1081. 10.1016/j.jmb.2005.12.003 16406067

[GR268490HARC61] Martin M. 2011. Cutadapt removes adapter sequences from high-throughput sequencing reads. EMBnet.journal 17: 10–12. 10.14806/ej.17.1.200

[GR268490HARC62] McMahon SB. 2014. MYC and the control of apoptosis. Cold Spring Harb Perspect Med 4: a014407. 10.1101/cshperspect.a014407 24985130 PMC4066641

[GR268490HARC63] Meyer C, Hofmann J, Burmeister T, Gröger D, Park TS, Emerenciano M, Pombo de Oliveira M, Renneville A, Villarese P, Macintyre E, 2013. The MLL recombinome of acute leukemias in 2013. Leukemia 27: 2165–2176. 10.1038/leu.2013.135 23628958 PMC3826032

[GR268490HARC64] Meyers RM, Bryan JG, McFarland JM, Weir BA, Sizemore AE, Xu H, Dharia NV, Montgomery PG, Cowley GS, Pantel S, 2017. Computational correction of copy-number effect improves specificity of CRISPR-Cas9 essentiality screens in cancer cells. Nat Genet 49: 1779–1784. 10.1038/ng.3984 29083409 PMC5709193

[GR268490HARC65] Milne TA. 2017. Mouse models of MLL leukemia: recapitulating the human disease. Blood 129: 2217–2223. 10.1182/blood-2016-10-691428 28179274 PMC5399479

[GR268490HARC66] Milne TA, Martin ME, Brock HW, Slany RK, Hess JL. 2005. Leukemogenic MLL fusion proteins bind across a broad region of the *Hox a9* locus, promoting transcription and multiple histone modifications. Cancer Res 65: 11367–11374. 10.1158/0008-5472.CAN-05-1041 16357144

[GR268490HARC67] Milo R, Shen-Orr S, Itzkovitz S, Kashtan N, Chklovskii D, Alon U. 2002. Network motifs: simple building blocks of complex networks. Science 298: 824–827. 10.1126/science.298.5594.824 12399590

[GR268490HARC68] Mueller D, Bach C, Zeisig D, Garcia-Cuellar MP, Monroe S, Sreekumar A, Zhou R, Nesvizhskii A, Chinnaiyan A, Hess JL, 2007. A role for the MLL fusion partner ENL in transcriptional elongation and chromatin modification. Blood 110: 4445–4454. 10.1182/blood-2007-05-090514 17855633 PMC2234781

[GR268490HARC69] Niu X, Wang G, Wang Y, Caldwell JT, Edwards H, Xie C, Taub JW, Li C, Lin H, Ge Y. 2014. Acute myeloid leukemia cells harboring MLL fusion genes or with the acute promyelocytic leukemia phenotype are sensitive to the Bcl-2-selective inhibitor ABT-199. Leukemia 28: 1557–1560. 10.1038/leu.2014.72 24531733 PMC4090260

[GR268490HARC70] O'Byrne S, Elliott N, Rice S, Buck G, Fordham N, Garnett C, Godfrey L, Crump NT, Wright G, Inglott S, 2019. Discovery of a CD10-negative B-progenitor in human fetal life identifies unique ontogeny-related developmental programs. Blood 134: 1059–1071. 10.1182/blood.2019001289 31383639

[GR268490HARC71] Okada Y, Feng Q, Lin Y, Jiang Q, Li Y, Coffield VM, Su L, Xu G, Zhang Y. 2005. hDOT1L links histone methylation to leukemogenesis. Cell 121: 167–178. 10.1016/j.cell.2005.02.020 15851025

[GR268490HARC72] Okuda H, Kanai A, Ito S, Matsui H, Yokoyama A. 2015. AF4 uses the SL1 components of RNAP1 machinery to initiate MLL fusion- and AEP-dependent transcription. Nat Commun 6: 8869. 10.1038/ncomms9869 26593443 PMC4673504

[GR268490HARC73] Orlando DA, Chen MW, Brown VE, Solanki S, Choi YJ, Olson ER, Fritz CC, Bradner JE, Guenther MG. 2014. Quantitative ChIP-Seq normalization reveals global modulation of the epigenome. Cell Rep 9: 1163–1170. 10.1016/j.celrep.2014.10.018 25437568

[GR268490HARC74] Pan R, Hogdal LJ, Benito JM, Bucci D, Han L, Borthakur G, Cortes J, DeAngelo DJ, Debose L, Mu H, 2014. Selective BCL-2 inhibition by ABT-199 causes on-target cell death in acute myeloid leukemia. Cancer Discov 4: 362–375. 10.1158/2159-8290.CD-13-0609 24346116 PMC3975047

[GR268490HARC75] Pillai V, Muralidharan K, Meng W, Bagashev A, Oldridge DA, Rosenthal J, Van Arnam J, Melenhorst JJ, Mohan D, DiNofia AM, 2019. CAR T-cell therapy is effective for CD19-dim B-lymphoblastic leukemia but is impacted by prior blinatumomab therapy. Blood Advances 3: 3539–3549. 10.1182/bloodadvances.2019000692 31738832 PMC6880911

[GR268490HARC76] Prange KHM, Mandoli A, Kuznetsova T, Wang SY, Sotoca AM, Marneth AE, van der Reijden BA, Stunnenberg HG, Martens JHA. 2017. MLL-AF9 and MLL-AF4 oncofusion proteins bind a distinct enhancer repertoire and target the RUNX1 program in 11q23 acute myeloid leukemia. Oncogene 36: 3346–3356. 10.1038/onc.2016.488 28114278 PMC5474565

[GR268490HARC77] Quinlan AR, Hall IM. 2010. BEDTools: a flexible suite of utilities for comparing genomic features. Bioinformatics 26: 841–842. 10.1093/bioinformatics/btq033 20110278 PMC2832824

[GR268490HARC78] Ramírez F, Ryan DP, Grüning B, Bhardwaj V, Kilpert F, Richter AS, Heyne S, Dündar F, Manke T. 2016. deepTools2: a next generation web server for deep-sequencing data analysis. Nucleic Acids Res 44: W160–W165. 10.1093/nar/gkw257 27079975 PMC4987876

[GR268490HARC79] R Core Team. 2021. R: a language and environment for statistical computing. R Foundation for Statistical Computing, Vienna. https://www.R-project.org/.

[GR268490HARC80] Reik W. 2007. Stability and flexibility of epigenetic gene regulation in mammalian development. Nature 447: 425–432. 10.1038/nature05918 17522676

[GR268490HARC81] Reiter F, Wienerroither S, Stark A. 2017. Combinatorial function of transcription factors and cofactors. Curr Opin Genet Dev 43: 73–81. 10.1016/j.gde.2016.12.007 28110180

[GR268490HARC82] Robinson BW, Behling KC, Gupta M, Zhang AY, Moore JS, Bantly AD, Willman CL, Carroll AJ, Adamson PC, Barrett JS, 2008. Abundant anti-apoptotic BCL-2 is a molecular target in leukaemias with t(4;11) translocation. Br J Haematol 141: 827–839. 10.1111/j.1365-2141.2008.07100.x 18422996

[GR268490HARC83] Robinson MD, McCarthy DJ, Smyth GK. 2010. edgeR: a Bioconductor package for differential expression analysis of digital gene expression data. Bioinformatics 26: 139–140. 10.1093/bioinformatics/btp616 19910308 PMC2796818

[GR268490HARC84] Rosenfeld N, Alon U. 2003. Response delays and the structure of transcription networks. J Mol Biol 329: 645–654. 10.1016/S0022-2836(03)00506-0 12787666

[GR268490HARC85] Sadelain M, Papapetrou EP, Bushman FD. 2012. Safe harbours for the integration of new DNA in the human genome. Nat Rev Cancer 12: 51–58. 10.1038/nrc3179 22129804

[GR268490HARC86] Slany RK. 2020. MLL fusion proteins and transcriptional control. Biochim Biophys Acta Gene Regul Mech 1863: 194503. 10.1016/j.bbagrm.2020.194503 32061883

[GR268490HARC87] Slee EA, Harte MT, Kluck RM, Wolf BB, Casiano CA, Newmeyer DD, Wang HG, Reed JC, Nicholson DW, Alnemri ES, 1999. Ordering the cytochrome c-initiated caspase cascade: hierarchical activation of caspases-2, -3, -6, -7, -8, and -10 in a caspase-9-dependent manner. J Cell Biol 144: 281–292. 10.1083/jcb.144.2.281 9922454 PMC2132895

[GR268490HARC88] Souers AJ, Leverson JD, Boghaert ER, Ackler SL, Catron ND, Chen J, Dayton BD, Ding H, Enschede SH, Fairbrother WJ, 2013. ABT-199, a potent and selective BCL-2 inhibitor, achieves antitumor activity while sparing platelets. Nat Med 19: 202–208. 10.1038/nm.3048 23291630

[GR268490HARC89] Takahashi S, Yokoyama A. 2020. The molecular functions of common and atypical MLL fusion protein complexes. Biochim Biophys Acta Gene Regul Mech 1863: 194548. 10.1016/j.bbagrm.2020.194548 32320750

[GR268490HARC90] Telenius J, The WIGWAM Consortium, Hughes JR. 2018. NGseqBasic - a single-command UNIX tool for ATAC-seq, DNaseI-seq, Cut-and-Run, and ChIP-seq data mapping, high-resolution visualisation, and quality control. bioRxiv 10.1101/393413

[GR268490HARC91] Thomas PD, Campbell MJ, Kejariwal A, Mi H, Karlak B, Daverman R, Diemer K, Muruganujan A, Narechania A. 2003. PANTHER: a library of protein families and subfamilies indexed by function. Genome Res 13: 2129–2141. 10.1101/gr.772403 12952881 PMC403709

[GR268490HARC92] Thomas M, Geßner A, Vornlocher HP, Hadwiger P, Greil J, Heidenreich O. 2005. Targeting MLL-AF4 with short interfering RNAs inhibits clonogenicity and engraftment of t(4;11)-positive human leukemic cells. Blood 106: 3559–3566. 10.1182/blood-2005-03-1283 16046533

[GR268490HARC93] Thoms JAI, Beck D, Pimanda JE. 2019. Transcriptional networks in acute myeloid leukemia. Genes Chromosomes Cancer 58: 859–874. 10.1002/gcc.22794 31369171

[GR268490HARC94] Tzelepis K, Koike-Yusa H, De Braekeleer E, Li Y, Metzakopian E, Dovey OM, Mupo A, Grinkevich V, Li M, Mazan M, 2016. A CRISPR dropout screen identifies genetic vulnerabilities and therapeutic targets in acute myeloid leukemia. Cell Rep 17: 1193–1205. 10.1016/j.celrep.2016.09.079 27760321 PMC5081405

[GR268490HARC95] Vandenberg CJ, Cory S. 2013. ABT-199, a new Bcl-2–specific BH3 mimetic, has in vivo efficacy against aggressive Myc-driven mouse lymphomas without provoking thrombocytopenia. Blood 121: 2285–2288. 10.1182/blood-2013-01-475855 23341542 PMC3606065

[GR268490HARC96] Wilkinson AC, Ballabio E, Geng H, North P, Tapia M, Kerry J, Biswas D, Roeder RG, Allis CD, Melnick A, 2013. *RUNX1* is a key target in t(4;11) leukemias that contributes to gene activation through an AF4-MLL complex interaction. Cell Rep 3: 116–127. 10.1016/j.celrep.2012.12.016 23352661 PMC3607232

[GR268490HARC97] Williams RM, Candido-Ferreira I, Repapi E, Gavriouchkina D, Senanayake U, Ling ITC, Telenius J, Taylor S, Hughes J, Sauka-Spengler T. 2019. Reconstruction of the global neural crest gene regulatory network *in vivo*. Dev Cell 51: 255–276.e7. 10.1016/j.devcel.2019.10.003 31639368 PMC6838682

[GR268490HARC98] Wilson NK, Timms RT, Kinston SJ, Cheng YH, Oram SH, Landry JR, Mullender J, Ottersbach K, Gottgens B. 2010. Gfi1 expression is controlled by five distinct regulatory regions spread over 100 kilobases, with Scl/Tal1, Gata2, PU.1, Erg, Meis1, and Runx1 acting as upstream regulators in early hematopoietic cells. Mol Cell Biol 30: 3853–3863. 10.1128/MCB.00032-10 20516218 PMC2916401

[GR268490HARC99] Yokoyama A, Lin M, Naresh A, Kitabayashi I, Cleary ML. 2010. A higher-order complex containing AF4 and ENL family proteins with P-TEFb facilitates oncogenic and physiologic MLL-dependent transcription. Cancer Cell 17: 198–212. 10.1016/j.ccr.2009.12.040 20153263 PMC2824033

[GR268490HARC100] Zuber J, Shi J, Wang E, Rappaport AR, Herrmann H, Sison EA, Magoon D, Qi J, Blatt K, Wunderlich M, 2011. RNAi screen identifies Brd4 as a therapeutic target in acute myeloid leukaemia. Nature 478: 524–528. 10.1038/nature10334 21814200 PMC3328300

