# Supplementary material for: A KMT2A-AFF1 gene regulatory network highlights the role of core transcription factors and reveals the regulatory logic of key downstream target genes
Source: Genome Res. 2021 Jul;31(7):1159–73. doi: 10.1101/gr.268490.120 (PMC8256865; doi:10.1101/gr.268490.120)
Supplement: Supplemental Material [file supp_31_7_1159__DC1.html]

A KMT2A-AFF1 gene regulatory network highlights the role of core transcription factors and reveals the regulatory logic of key downstream target genes — Supplemental Material 

# A KMT2A-AFF1 gene regulatory network highlights the role of core transcription factors and reveals the regulatory logic of key downstream target genes

## Supplemental Material

- Supplemental\_Material.pdf
- Supplemental\_Code.zip
- Supplemental\_Data\_S1.xlsx
- Supplemental\_Data\_S2.xlsx
- Supplemental\_Data\_S3.xlsx
- Supplemental\_Data\_S4.xlsx
- Supplemental\_Data\_S5.xlsx
