# Supplementary material for: A KMT2A-AFF1 gene regulatory network highlights the role of core transcription factors and reveals the regulatory logic of key downstream target genes
Source: Genome Res. 2021 Jul;31(7):1159–73. doi: 10.1101/gr.268490.120 (PMC8256865; doi:10.1101/gr.268490.120)
Supplement: Supplemental Material [file supp_gr.268490.120_Supplemental_Material.pdf]

SUPPLEMENTAL INFORMATION

A KMT2A-AFF1 gene regulatory network highlights the role of core transcription factors and reveals the regulatory logic of key downstream target genes.

Joe R. Harman[1,†], Ross Thorne[1,†], Max Jamilly[2], Marta Tapia[1,3], Nicholas T. Crump[1], Siobhan Rice[1,4], Ryan Beveridge[1,5], Edward Morrissey[6], Marella F.T.R. de Bruijn[1], Irene Roberts[4,8], Anindita Roy[4,8], Tudor A. Fulga[2,7], Thomas A. Milne[1,8*]

<u>Supplemental items:</u>

Supplemental Fig S1 (Page 2). KMT2A-N binding profiles and input tracks in SEM and patient data.

Supplemental Fig S2 (Page 3). Comparing the SEM KMT2A-AFF1 GRN with RS4;11 KMT2A-AFF1, and SEM DOT1L and BRD4 networks.

Supplemental Fig S3 (Page 4). Patient sub-network analysis highlights core transcription factors.

Supplemental Fig S4 (Page 5). KMT2A-AFF1 and RUNX1 binding shows similarities in KMT2A-AFF1 ALL and KMT2A-MLLT3 AML models.

Supplemental Fig S5 (Page 6). Comparison of KMT2A-AFF1 GRN nodes with published CRISPR essentiality screens.

Supplemental Fig S6 (Page 7). KMT2A-AFF1 cooperates with RUNX1 in FFL and cascade circuits to regulate downstream targets.

Supplemental Fig S7 (Page 8). CRISPR screen in combination with venetoclax treatment to test GRN predicted circuits.

Supplemental Fig S8 (Page 9). KMT2A-AFF1 and RUNX1 cooperate to regulate *CASP9* in a cascade motif.

Supplemental Table S1 (Page 10). List of antibodies.

Supplemental Table S2 (Page 11). List of primers.

Supplemental Table S3 (Page 12). GEO accession numbers for previously published sequencing experiments.

Supplemental Data S1. KMT2A-AFF1 and RUNX1 network annotated edge and node tables.

Supplemental Data S2. Patient sub-network node clusters.

Supplemental Data S3. Nascent RNA-seq results and PANTHER pathway enrichment.

Supplemental Data S4. KMT2A-AFF1 – RUNX1 regulatory FFL and cascade circuits.

Supplemental Data S5. Results of CRISPR screen in combination with venetoclax treatment in THP-1 cells.

Supplemental Code. Jupyter Notebook files of custom scripts used in the study.

Figure S1

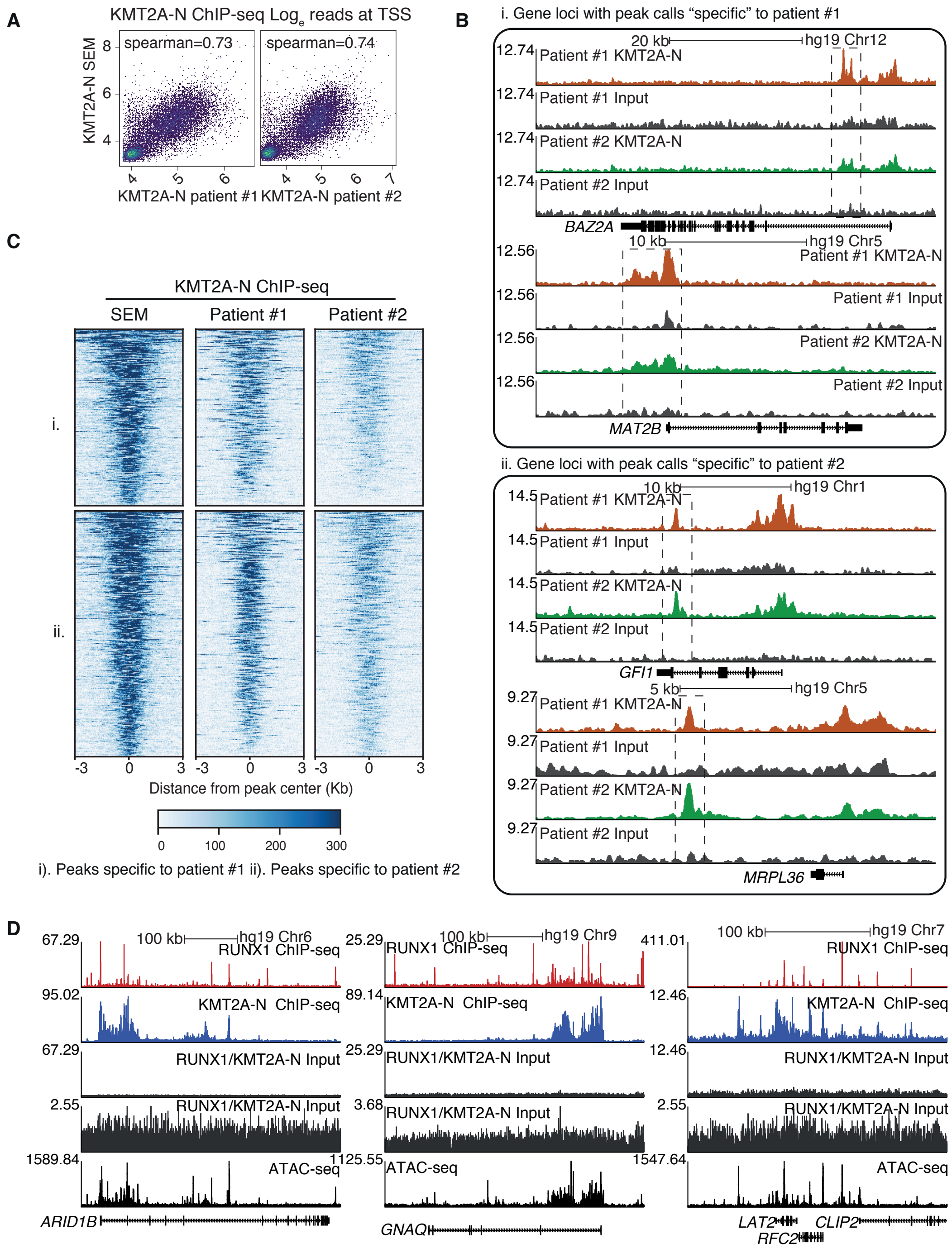

Supplemental Fig S1. KMT2A-N binding profiles and input tracks in SEM and patient data. (*A*) Scatter plot showing Log_e KMT2A-N reads at TSS in SEM cells compared with patient samples #1 and #2. (*B*) ChIP-seq tracks generated in hg19 for KMT2A-N, reads normalized to $1{\times}10^7$ reads. Loci are KMT2A-AFF1 bound genes unique to patient #1 (i) or patient #2 (ii), as defined by peak calling as shown in Fig. 1B. (*C*) Heatmaps of KMT2A-N ChIP-seq signal in SEM cells, patient #1 and patient #2. Heatmaps generated over KMT2A-N peaks with peak calls unique to patient #1 (i) or patient #2 (ii). (*D*) ChIP-seq tracks generated in hg19 for KMT2A-N, RUNX1, and input chromatin. Input tracks are shown scaled to maximum signal at the locus, or matching track height in RUNX1 or KMT2A-N (whichever is lowest). ATAC-seq is also included to show accessible regions. ChIP-seq and ATAC-seq data normalized to $1{\times}10^7$ reads. Displayed loci are KMT2A-AFF1 and RUNX1 targets.

Figure S2

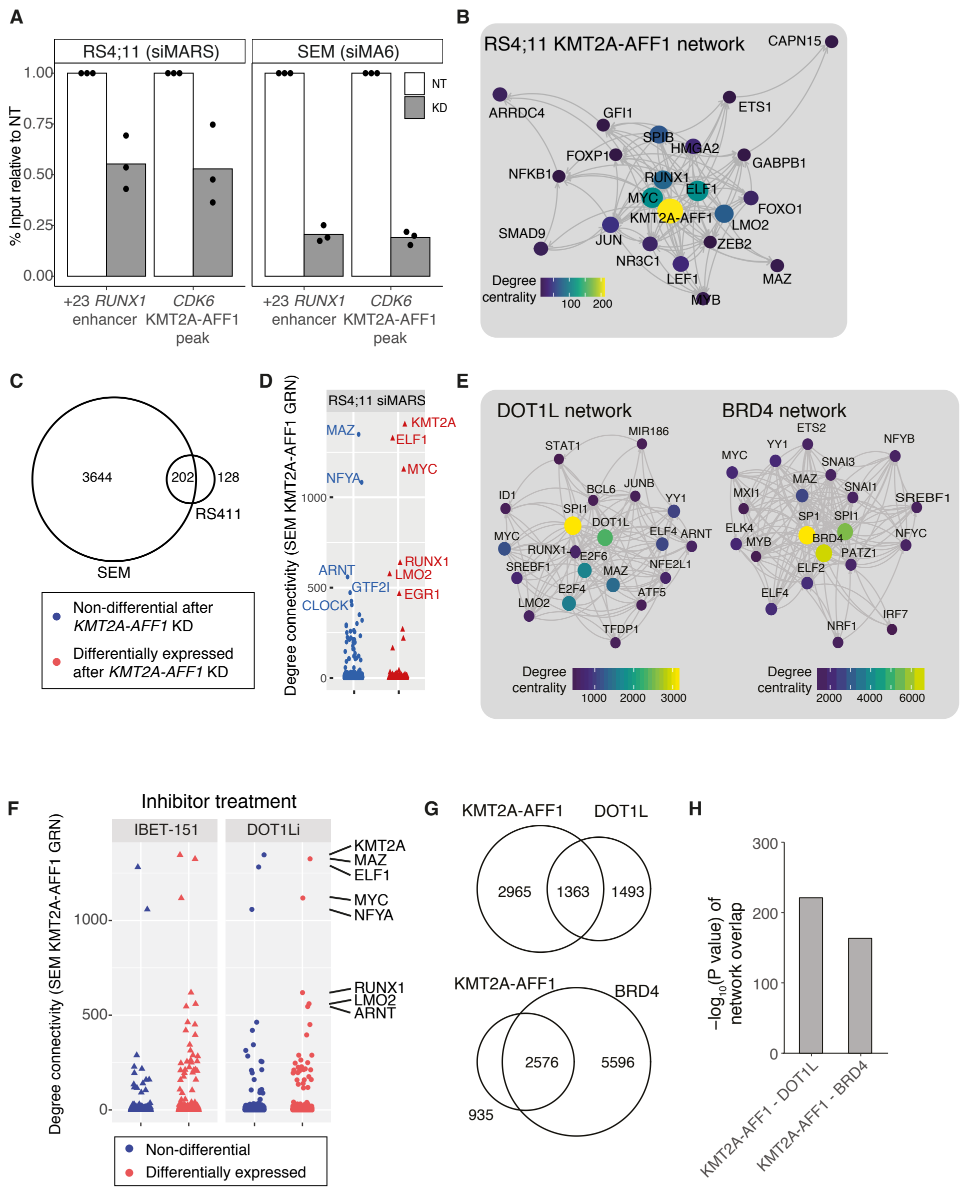

Supplemental Fig S2. Comparing the SEM KMT2A-AFF1 GRN with RS4;11 KMT2A-AFF1, and SEM DOT1L and BRD4 networks. (*A*) KMT2A-N ChIP qPCR data in RS4;11 and SEM cells after *KMT2A-AFF1* KD, normalized to input chromatin and displayed relative to NT control, at the *RUNX1* +23 enhancer and an KMT2A-AFF1 peak at the *CDK6* locus. siMA6 and siMARS refers to SEM and RS4;11 specific MLL-AF4 siRNA, respectively. (*B*) Top 20 genes of the RS4;11 KMT2A-AFF1 GRN by degree centrality. Lines indicate predicted interaction from protein to gene locus, with arrowheads pointing towards downstream nodes. (*C*) Overlap of nodes between SEM and RS4;11 KMT2A-AFF1 GRN models. (*D*) DEGs after *KMT2A-AFF1* KD in RS4;11 cells (n=3, FDR <0.05), plotted against degree centrality in SEM KMT2A-AFF1 GRN. (*E*) Top 20 genes of the DOT1L (left) and BRD4 (right) GRN models by degree centrality. Lines indicate predicted interaction from protein to gene locus, with arrowheads pointing downstream. (*F*) DEGs upon 1.5 hours’ IBET or 7 days’ EPZ-5676 treatment, plotted against degree centrality in SEM KMT2A-AFF1 GRN (n=3). (*G*) Overlap of nodes between KMT2A-AFF1, DOT1L and BRD4 GRNs. (*H*) Statistical significance of overlaps shown in (G). Significance calculated using a Fisher’s exact test and displayed as -$\log_{10}$(P value).

Figure S3

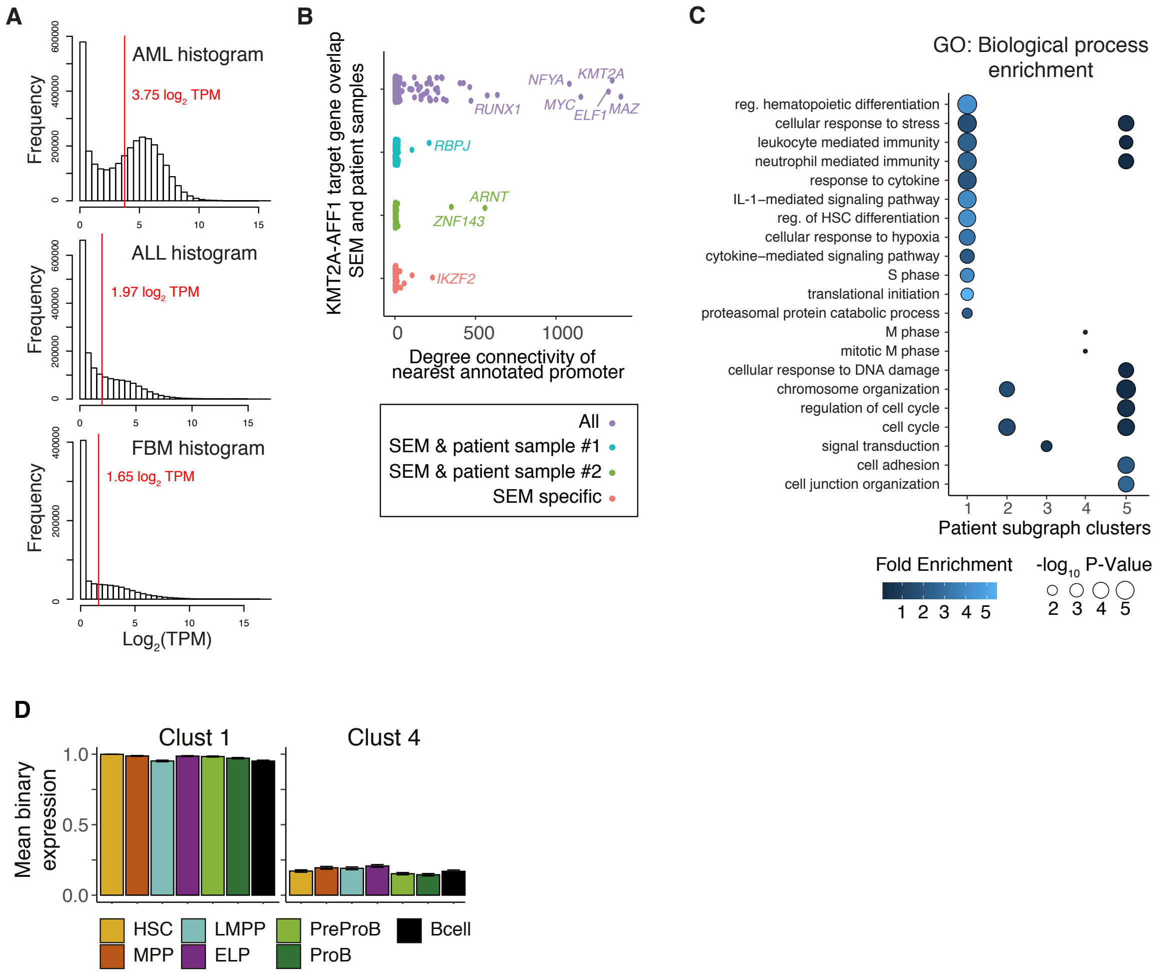

Supplemental Fig S3. Patient sub-network analysis highlights core transcription factors. (*A*) Histograms of $\log_2$ TPM expression across AML, ALL and FBM datasets. Red line indicates mean $\log_2$ TPM for each dataset, the threshold used to define active expression as used in generating patient-specific sub-networks (see Figure 2). (*B*) Overlap of SEM KMT2A-AFF1 bound genes with genes bound in patient #1 and #2, as in Figure 1B, against connectivity within the SEM KMT2A-AFF1 GRN. (*C*) GO Biological process enrichment for patient subgraph clusters. Size of points represents $-\log_{10}$ FDR of enrichment, while point color represents fold enrichment over expected. (*D*) Bar plots of FBM cell populations showing mean binary expression in clusters 1 and 4.

Figure S4

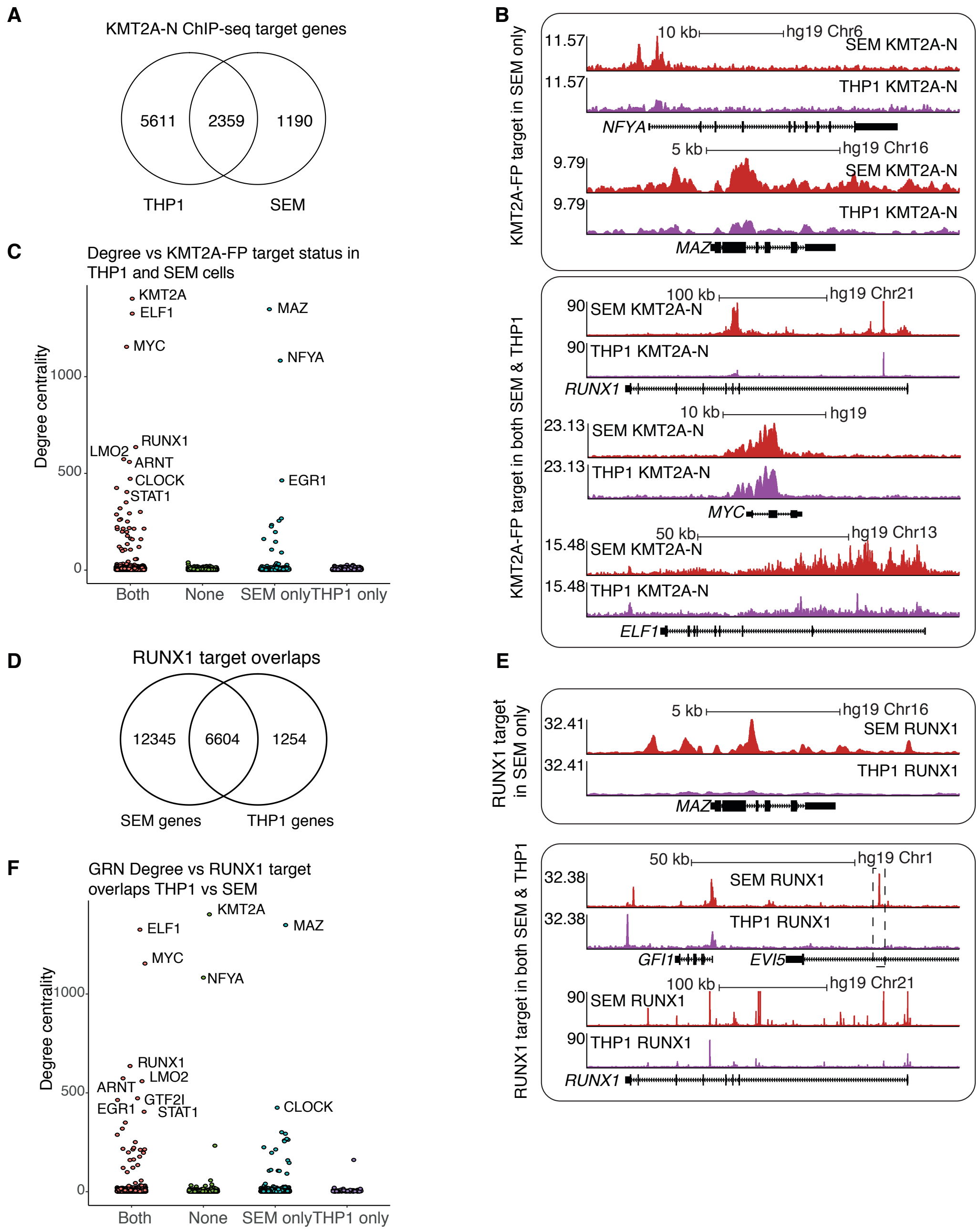

Supplemental Fig S4. KMT2A-FP and RUNX1 binding show similarities in KMT2A-AFF1 ALL and KMT2A-MLLT3 AML models. (*A*) Overlap of KMT2A-N bound genes in THP-1 and SEM cells. (*B*) ChIP-seq tracks generated in hg19 for KMT2A-N in SEM and THP-1 cells. Reads normalized to $1{\times}10^7$ reads. Displayed loci are KMT2A-FP bound genes unique to SEM, or common to both SEM and THP-1. (*C*) Association of Log_2 degree centrality with KMT2A-N bound gene overlaps in THP-1 and SEM cells as shown in (A). (*D*) Overlap of RUNX1 bound genes in THP-1 and SEM cells. (*E*) ChIP-seq tracks generated in hg19 for RUNX1 in SEM and THP-1 cells. Reads normalized to $1{\times}10^7$ reads. Displayed loci are RUNX1 bound genes unique to SEM, or common to both SEM and THP-1. Dashed box highlights an SEM specific RUNX1 peak. (*F*) Association of Log_2 degree centrality with RUNX1 target overlaps in THP-1 and SEM cells as shown in (D).

Figure S5

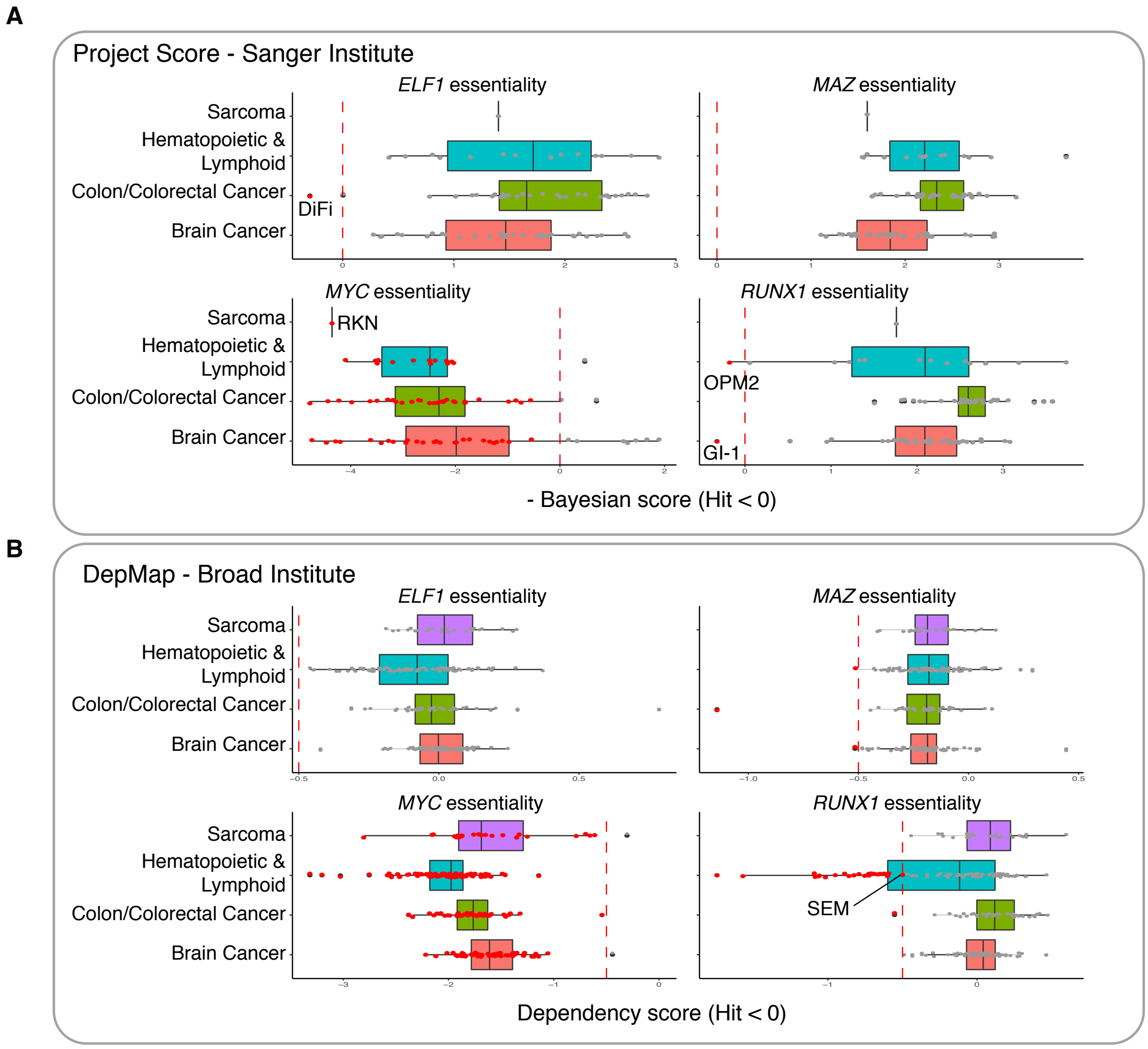

Supplemental Fig S5. Comparison of KMT2A-AFF1 GRN nodes with published CRISPR essentiality screens. (*A*) and (*B*) CRISPR essentiality screen for *RUNX1*, *MYC*, *MAZ* and *ELF1* from (A) the Project Score database of the Sanger Institute Cancer Dependency Map (Behan et al. 2019), and (B) the Avana 21Q1 dataset from the Broad Institute Cancer Dependency Map (Meyers et al. 2017; Doench et al. 2016). For Project Score, inverted Bayesian show essential hits below 0, and for the Avana dataset CERES scores show essential hits below 0.5. Each datapoint represents a different cell line, with essentiality highlighted in red. Cell lines were categorized into the type of cancer that they model.

Figure S6

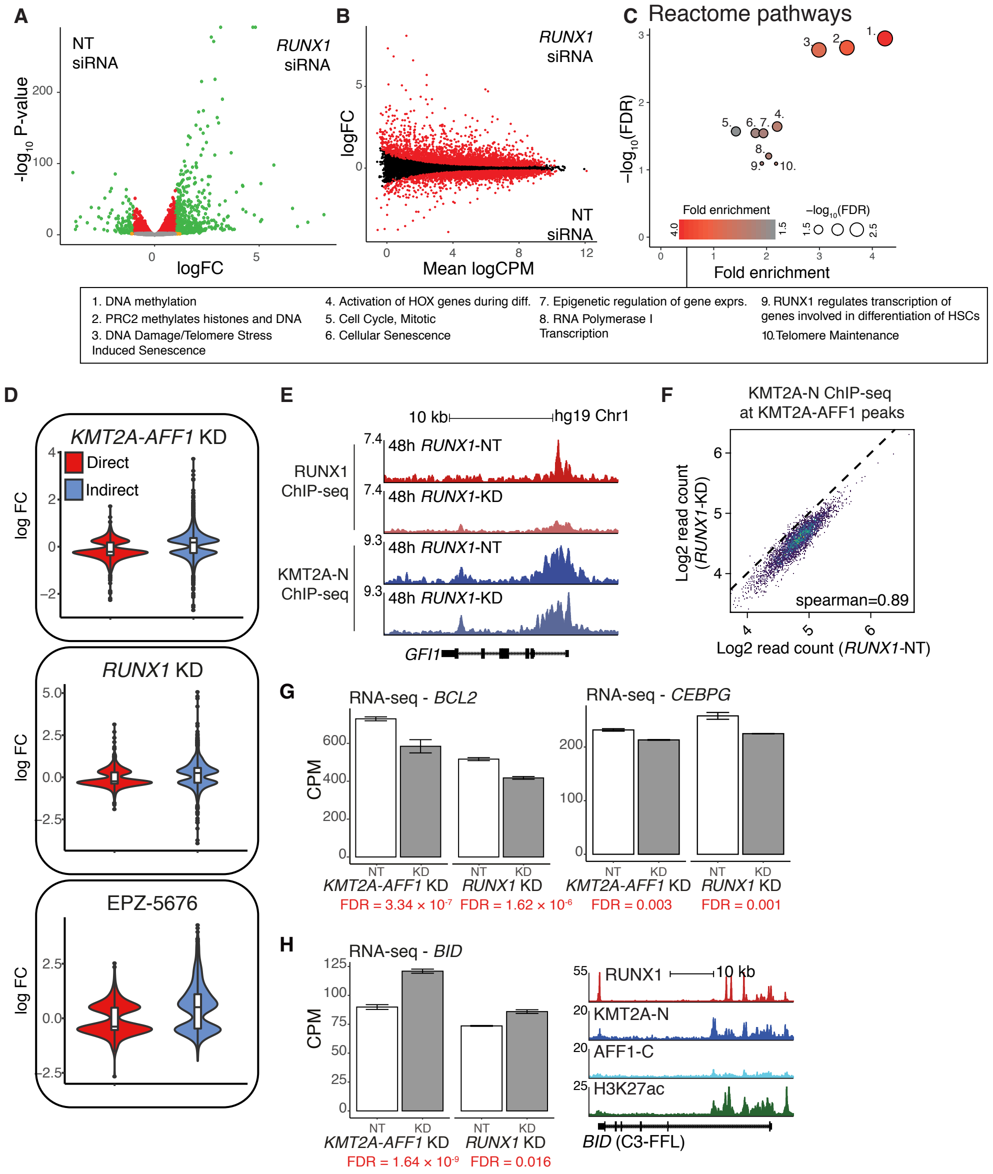

Supplemental Fig S6. KMT2A-AFF1 cooperates with RUNX1 in FFL and cascade circuits to regulate downstream targets. (*A*) Volcano plot showing the relationship between -$\log_{10}$ P value and |logFC| in *RUNX1* KD nascent RNA-seq. Orange points represent genes |logFC| ≥ 1 and FDR ≥ 0.05; red represents |logFC| < 1 and FDR < 0.05; green represents |logFC| ≥ 1 and FDR < 0.05. (*B*) MA plot showing the relationship between logFC and mean $\log_2$ CPM in *RUNX1* KD nascent RNA-seq. DEGs (FDR < 0.05) are highlighted in red. (*C*) Pathway enrichment (Reactome) for overlap between *KMT2A-AFF1* and *RUNX1* KD DEGs (Figure 5B). Size of points represents -$\log_{10}$ FDR of enrichment, while point color represents fold enrichment over expected number of genes. (*D*) Violin and boxplots showing logFC expression response to *KMT2A-AFF1* KD, *RUNX1* KD, or EPZ-5676 . Data split between genes in the direct KMT2A-AFF1 GRN (genes directly bound by KMT2A-AFF1) and the indirect GRN (genes not bound by KMT2A-AFF1). (*E*) Reference-normalized ChIP-seq tracks generated in hg19 for KMT2A-N and RUNX1 after 48 hours' *RUNX1* KD. Reads normalized to $1{\times}10^7$ reads. (*F*) Scatter plot showing KMT2A-N reads at KMT2A-AFF1 peaks following 48 hours' treatment with NT or *RUNX1* siRNA. (*G*) Expression of *BCL2* and *CEBPG* from nascent RNA-seq data following KMT2A-AFF1 or *RUNX1* KD. Expression normalized as CPM. (*H*) Left - Expression of *BID* from nascent RNA-seq data following *KMT2A-AFF1* or *RUNX1* KD. Expression normalized as CPM. Right - ChIP-seq tracks generated in hg19 for KMT2A-N, AFF1-C, RUNX1 and H3K27ac at *BID* locus. Reads normalized to $1{\times}10^7$ reads.

Figure S7

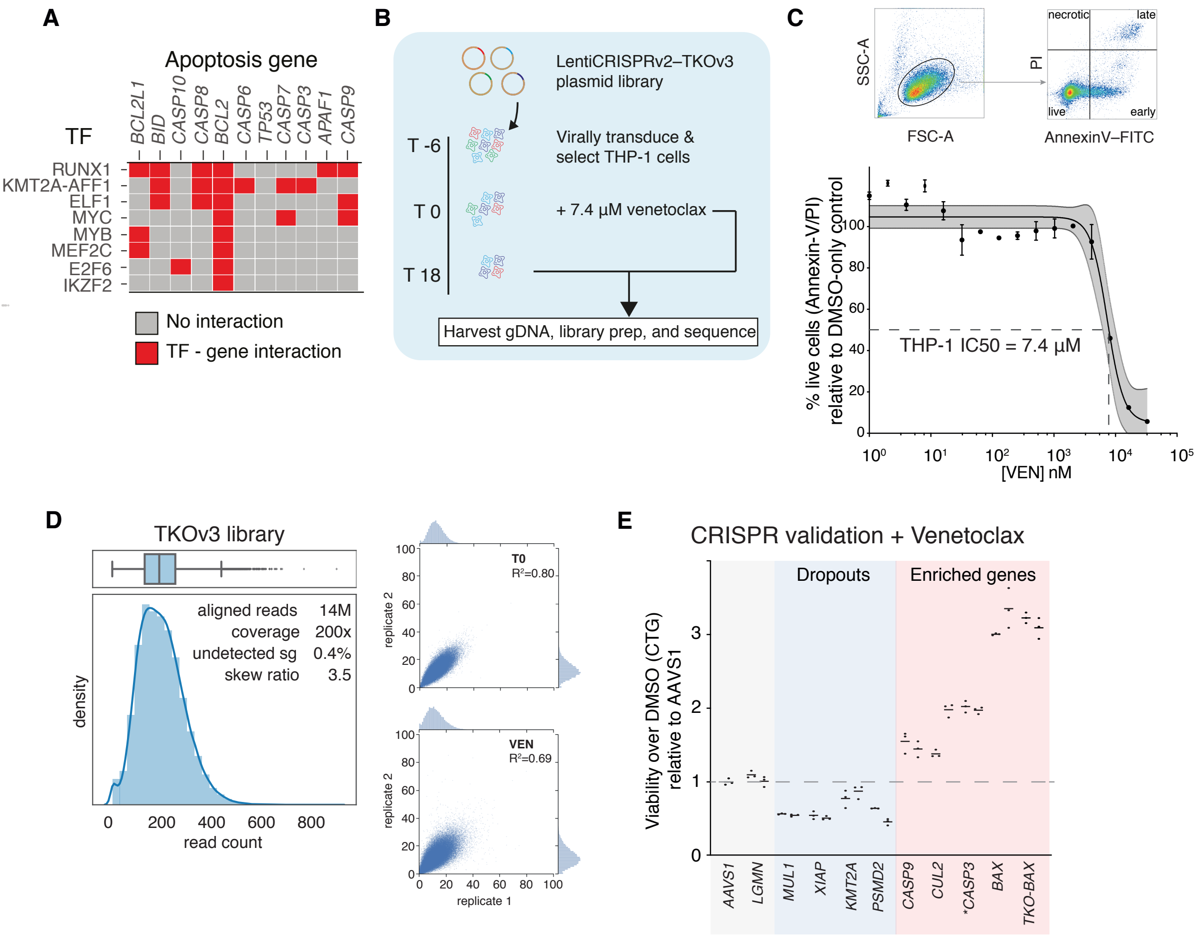

Supplemental Fig S7. CRISPR screen in combination with venetoclax treatment to test GRN predicted circuits. (*A*) Example interaction matrix showing GRN predicted TF regulation of the apoptosis pathway. Red squares indicate a predicted regulatory interaction between the TF and apoptosis gene. (*B*) Experimental outline of CRISPR screen performed in combination with 7.4 μM venetoclax treatment in THP-1 cells. Cells harvested for sgRNA counting and analysis at T0 (after transduction and selection) and T18 (18 days treatment with venetoclax). (*C*) Above – Flow cytometry gating for Annexin-V/PI staining of THP-1 cells to assay viability with venetoclax treatment. Below – THP-1 cells were cultured for 48 hours with different concentrations of venetoclax, and assayed for viability. PI: propidium iodide; early/late: early/late apoptotic. Dots show mean; error bars show SD. Lines show unconstrained four parameter sigmoidal least-squares regression best fit; shaded region shows 99% confidence interval. For all curves, $R^2 > 0.99$. (n=5). (*D*) Left – Sequencing validation of amplified sgRNA pool showing sgRNA distribution as a histogram and boxplot. Right – Scatter plot of sgRNA counts for biological replicates of T0 and T18+VEN samples. Pearson correlation between replicates shown as R^2, and histograms distributions shown along the axis. (*E*) Functional validation of CRISPR screen hits by individual gene knockouts using sgRNA from the Brunello library. sgRNA were cloned into lentiCRISPRv2 constructs and transduced into THP-1 cells, followed by 48 hours' treatment with DMSO or 20 μM venetoclax. CellTiter Glo (CTG) was used to assay viability, and is normalized as Venetoclax/DMSO viability, relative to AAVS1 silent control. Genes marked * are FDR > 0.1 in the CRISPR screen. Dots show biological replicates (n=3); bars show mean.

Figure S8

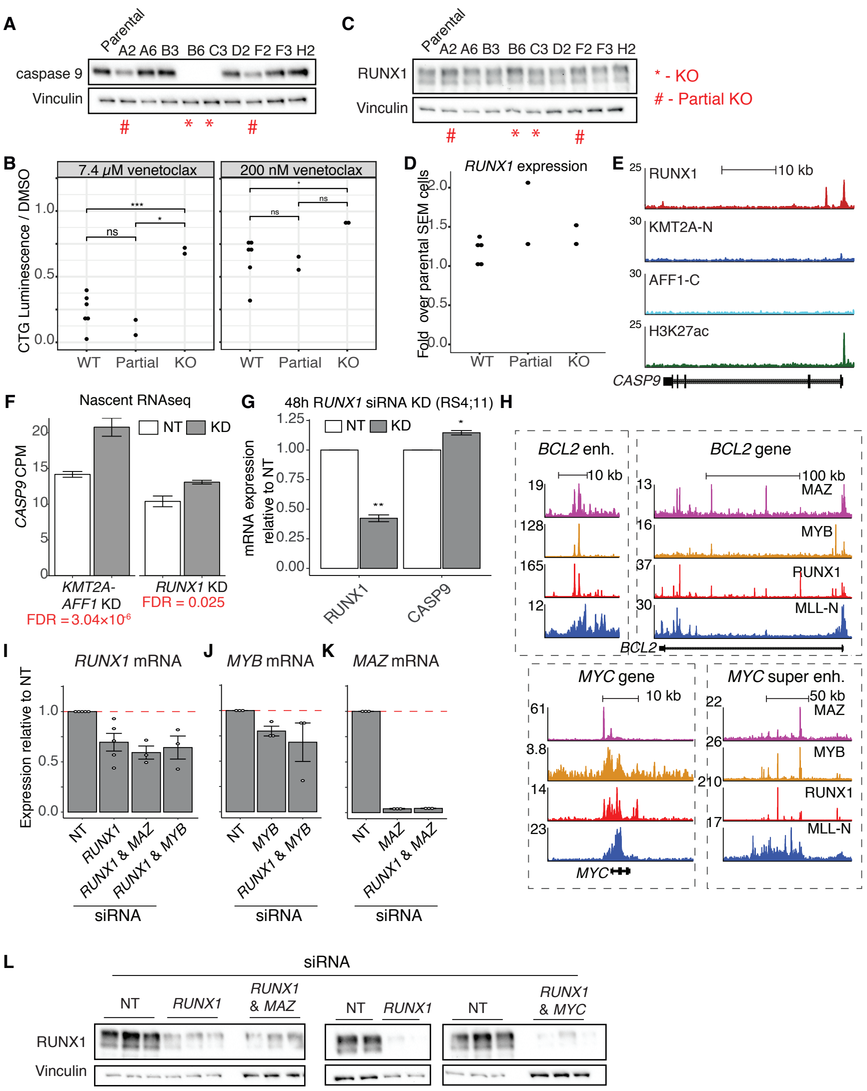

Supplemental Fig S8. KMT2A-AFF1 and RUNX1 cooperate to regulate *CASP9* in a cascade motif. (*A*) Western blot for caspase 9 in *CASP9* knockout clones in SEM cells. KO lines indicated with a *, and partial KO with #. Parental refers to original SEM line without clonal selection. (*B*) CTG viability assay of CASP9 KO clones following 48 hours' treatment with 7.4 μM venetoclax (left) or 200 nM venetoclax (IC50 in SEM cells (Benito et al. 2015) (right). CTG luminescence displayed as a ratio relative to DMSO control. Separate clones are used as biological replicates (WT n=5, KO and partial KO n=2). (*C*) Western blot for RUNX1 in *CASP9* KO clones, with vinculin as loading control. *CASP9* KO lines indicated with a *, and partial KO with #. (*D*) qRT-PCR results showing *RUNX1* expression relative to NT control in *CASP9* KO cells. (*E*) ChIP-seq tracks generated in hg19 for KMT2A-N, AFF1-C, RUNX1 and H3K27ac at *CASP9* locus. Reads normalized to $1{\times}10^7$ reads. (*F*) Expression of *CASP9* from nascent RNA-seq data following 96 hours' *KMT2A-AFF1* KD. Expression normalized as CPM. (*G*) qRT-PCR results showing *RUNX1* and *CASP9* expression relative to NT control after 48 hours' *RUNX1* KD in RS4;11 cells. (*H*) ChIP-seq tracks generated in hg19 for MAZ, MYB, RUNX1 and KMT2A-N. Visualized loci include and *BCL2* and *MYC* loci, as well as the *BCL2* enhancer (218 Kb downstream of promoter) and *MYC* super enhancer (1.8 Mb downstream of promoter). Reads normalized to $1{\times}10^7$ reads. (*I* - *K*) qRT-PCR analysis for *RUNX1* (I) and *MYB* (J) and *BCL2* (K) after 96 hours' siRNA treatment targeting genes as indicated (n=3, n=5 for RUNX1 KD). Expression normalized to mature *GAPDH* mRNA levels, and shown relative to NT control. (*L*) Western blot for RUNX1 after 96 hours' siRNA treatment targeting genes as indicated. Error bars represent standard error of the mean; * P < 0.05, ** P < 0.01.

Target	Application	Dilution	Catalogue number	Company
RUNX1	Western blotting	1/5,000	4334S	Cell Signaling
caspase 9	Western blotting	1/10,000	ab202068	Abcam
GAPDH	Western blotting	1/10,000	A300-641A	Bethyl
Vinculin	Western blotting	1/50,000	ab129002	Abcam
RUNX1	ChIP / ChIP-seq	1/500	ab23980	Abcam
KMT2A-N	ChIP / ChIP-seq	1/500	A300-086A	Bethyl
AFF1-C	ChIP-seq	1/500	ab31812	Abcam
MAZ	ChIP-seq	1/500	A301-652A	Bethyl

Supplementary Table S1. List of antibodies used in this study.

Target	Primer/probe	Note
GAPDH	Hs03929097_g1	TaqMan probe, qRT-PCR
RUNX1	Hs00231079_m1	TaqMan probe, qRT-PCR
CASP9	Hs00609647_m1	TaqMan probe, qRT-PCR
BCL2	Hs00608023_m1	TaqMan probe, qRT-PCR
MYC	Hs0015348_m1	TaqMan probe, qRT-PCR
BCL2 (intronic)	F - CGATAACGCCTGCCATCTAA<br>R - CCACCACATCCTACTGGATTAC	SYBR, qRT-PCR (pre-mRNA)
MYC (intronic)	F - AAGGGAGGCGAGGATGTGTCC<br>R - GGCTGGGTGCGGAGATTCG	SYBR, qRT-PCR (pre-mRNA)
KMT2A-AFF1 (SEM)	F - AGGTCCAGAGCAGAGCAAAC<br>R - CGGCCATGAATGGGTCATTTC	SYBR, qRT-PCR
KMT2A-AFF1 (RS4;11)	F - TCAGCACTCTCTCCAATGGCAATAG<br>R - GGGGTTTGTTCACTGTCACTGTCC	SYBR, qRT-PCR
Negative control locus	F - GGCTCCTGTAACCAACCACTACC<br>R - CCTCTGGGCTGGCTTCATTC	SYBR, ChIP-qPCR
+23 *RUNX1* enhancer	F - TGCGAGAGCGAGAAAACCACAG<br>R - GCAGAAAGCAACAGCCAGAAACG	SYBR, ChIP-qPCR
CDK6 KMT2A-AFF1 peak	F - TCGAAGCGAAGTCCTCAACA<br>R - GCTTGGGCAGAGGCTATGTA	SYBR, ChIP-qPCR

Supplementary Table S2. List of primers used in this study.

Experiment	Cell line/tissue	Antibody	Treatment	Accession Number
ChIP-seq	SEM	KMT2A-N		GSE74812
ChIP-seq	SEM	AFF1-C		GSE74812
ChIP-seq	SEM	RUNX1		GSE42075
ChIP-seq	SEM	Input		GSE42075
ChIP-seq	SEM	ELF1		GSE117865
ChIP-seq	SEM	H3K79me3		GSE117865
ChIP-seq	SEM	BRD4		GSE83671
ChIP-seq	SEM	H3K27ac		GSE74812
ChIP-seq	SEM	MYB		GSE117865
ATAC-seq	SEM			GSE74812
ChIP-seq	Primograft	KMT2A-N		GSE83671
ChIP-seq	Primograft	AFF1-C		GSE83671
Nascent RNA-seq	SEM		KMT2A-AFF1 siRNA	GSE85988
Nascent RNA-seq	SEM		EPZ-5676	GSE83671
Nascent RNA-seq	SEM		IBET	GSE139437

Supplementary Table S3. GEO accession numbers for previously published sequencing experiments
